# Phenotypic diversity of T cells in human primary and metastatic brain tumors revealed by multiomic interrogation

**DOI:** 10.1038/s43018-023-00566-3

**Published:** 2023-05-22

**Authors:** Vladimir Wischnewski, Roeltje R. Maas, Paola Guerrero Aruffo, Klara Soukup, Giovanni Galletti, Mara Kornete, Sabine Galland, Nadine Fournier, Johanna Lilja, Pratyaksha Wirapati, Joao Lourenco, Alice Scarpa, Roy T. Daniel, Andreas F. Hottinger, Jean-Philippe Brouland, Agnese Losurdo, Emanuele Voulaz, Marco Alloisio, Monika E. Hegi, Enrico Lugli, Johanna A. Joyce

**Affiliations:** 1 Department of Oncology, University of Lausanne, Lausanne, Switzerland; 2 Ludwig Institute for Cancer Research, University of Lausanne, Lausanne, Switzerland; 3 Agora Cancer Research Centre Lausanne, Lausanne, Switzerland; 4 Lundin Family Brain Tumor Research Center, Departments of Oncology and Clinical Neurosciences, Centre Hospitalier Universitaire Vaudois, Lausanne, Switzerland; 5 Neuroscience Research Center, Centre Hospitalier Universitaire Vaudois, Lausanne, Switzerland; 6 Department of Neurosurgery, Centre Hospitalier Universitaire Vaudois, Lausanne, Switzerland; 7 Laboratory of Translational Immunology, IRCCS Humanitas Research Hospital, Milan, Italy; 8 Department of Oncology, Centre Hospitalier Universitaire Vaudois, Lausanne, Switzerland; 9 Translational Data Science, Swiss Institute of Bioinformatics, Lausanne, Switzerland; 10 Department of Pathology, Centre Hospitalier Universitaire Vaudois, Lausanne, Switzerland; 11 Oncology Department, IRCCS Humanitas Research Hospital, Milan, Italy; 12 Department of Biomedical Sciences, Humanitas University, Milan, Italy; 13 Division of Thoracic Surgery, IRCCS Humanitas Research Hospital, Milan, Italy

**Keywords:** CNS cancer, Tumour immunology, Cancer

## Abstract

The immune-specialized environment of the healthy brain is tightly regulated to prevent excessive neuroinflammation. However, after cancer development, a tissue-specific conflict between brain-preserving immune suppression and tumor-directed immune activation may ensue. To interrogate potential roles of T cells in this process, we profiled these cells from individuals with primary or metastatic brain cancers via integrated analyses on the single-cell and bulk population levels. Our analysis revealed similarities and differences in T cell biology between individuals, with the most pronounced differences observed in a subgroup of individuals with brain metastasis, characterized by accumulation of *CXCL13*-expressing CD39^+^ potentially tumor-reactive T (pTRT) cells. In this subgroup, high pTRT cell abundance was comparable to that in primary lung cancer, whereas all other brain tumors had low levels, similar to primary breast cancer. These findings indicate that T cell-mediated tumor reactivity can occur in certain brain metastases and may inform stratification for treatment with immunotherapy.

## Abstract (web summary)

Joyce and colleagues use bulk and single-cell profiling of T cell phenotypes in human samples from primary brain tumors and brain metastases as a resource for understanding the biology and therapeutic relevance of the brain tumor microenvironment.

## Main

Malignancies in the brain are subdivided into primary tumors arising within this tissue, such as gliomas, and tumors of extracranial origin that subsequently metastasize to the brain^1^. Primary tumors most frequently associated with the development of brain metastasis (BrM) include lung, melanoma and breast cancers (BCs)^2^. Despite improvements in tumor detection and local treatment and the introduction of new therapies, including molecular-targeted and immune-based approaches, the prognosis for individuals diagnosed with aggressive brain cancer remains poor, with estimated 2-year survival rates of <20% (refs. ^2–4^), underscoring the need to better understand this disease as a means to develop effective therapies.

Although cytotoxic T cells are generally excluded from healthy brain parenchyma to prevent excessive neuroinflammation under homeostatic conditions, the impairment of the blood–brain barrier in advanced brain malignancies can facilitate the infiltration of peripheral immune cells, including CD8^+^ T cells^5,6^. The abundance of CD8^+^ T cells, however, is heterogeneous and typically higher in BrM than in gliomas^5,7^. Several clinical trials assessing the efficacy of immune checkpoint blockade (ICB) to target T cells have shown some intracranial responses in small subgroups of individuals with melanoma^8^ and lung^9^ cancer with BrM but have largely failed in individuals with high-grade gliomas^10^.

The heterogeneous efficacy of ICB in individuals with intracranial, but also extracranial, tumors has resulted in substantial efforts to identify biomarkers that would reliably predict response to this treatment. Together with tumor-intrinsic factors, including mismatch repair deficiency^11^ and mutational burden^12^, the presence of CD8^+^ tumor-infiltrating lymphocytes (TILs) is associated with better response to ICB and prolonged overall survival in extracranial tumors^13,14^ and BrM^15^.

Given the considerable phenotypic diversity of CD8^+^ T cells in tumors^16^, several studies have sought to identify distinct subsets associated with, and responsible for, the response to ICB. Clonal expansion^17,18^ and the expression of a distinct dysfunctional program in CD8^+^ T cells correlated with better outcome in several cancer types^19–21^. Moreover, RNA expression of *CXCL13* by clonally expanded CD8^+^ T cells with an exhaustion phenotype has emerged as a robust predictor for ICB efficacy across multiple tumor types^22–24^. Initial analyses have associated these CD8^+^ T cell subsets with reactivity against a subset of tumor antigens, suggesting direct tumor reactivity^25^. The abundance of these potentially tumor-reactive T (pTRT) cells varies substantially across cancer types, which may influence ICB response^26^. Critically, whether pTRT cells can also infiltrate brain tumors and, if so, to what extent remain unknown.

To address this question, we performed a comprehensive analysis of circulating and tumor-infiltrating T cells in a large cohort of 84 individuals with brain cancer (36 with glioma and 48 with BrM) and 44 individuals with extracranial tumors (33 lung primary tumors and 11 breast primary tumors). Our analyses included single-cell and bulk RNA sequencing (RNA-seq), T cell antigen receptor (TCR) profiling, high-dimensional flow cytometry (FCM) and immunofluorescence (IF) spatial imaging and functional ex vivo assays. This multifaceted orthogonal strategy revealed that a subgroup of individuals with BrM, but not glioma, displayed substantial tumor infiltration with pTRT cells. Notably, most of these pTRT cell-high BrM arose from lung cancer and showed comparable pTRT cell abundance to primary lung cancer samples. Together, these results indicate that T cell-mediated tumor reactivity within the brain can occur, albeit in a subset of individuals with BrM.

## Results

### Single-cell RNA-seq identifies pTRT cells in individuals with BrM

**Fig. 1:**
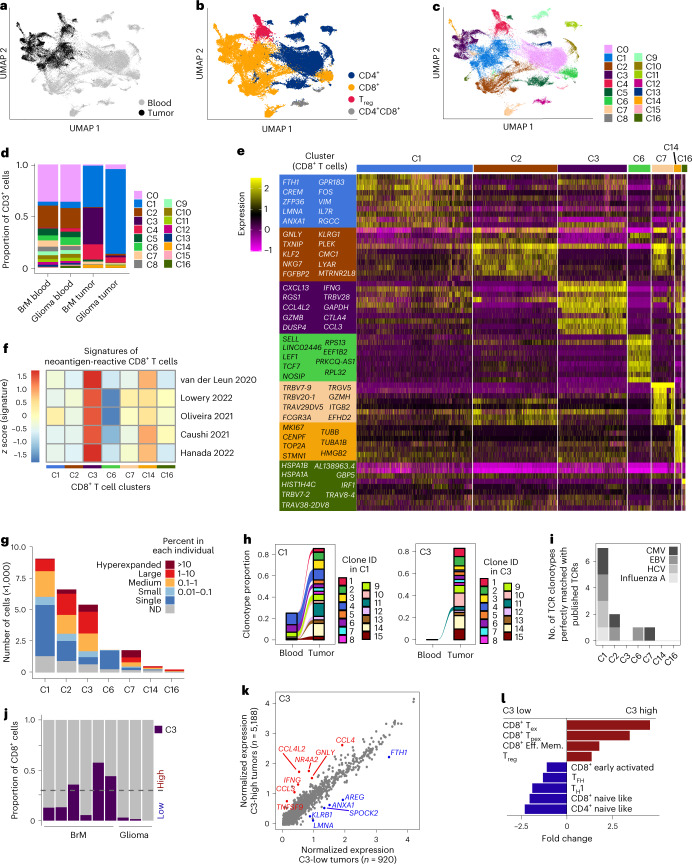
scRNA-seq identifies pTRT cells in a subset of individuals with BrM and defines a BrM pTRT cell-specific gene signature. **a**–**c**, UMAP of T cells from nine individuals with brain cancer colored by tissue (**a**), cell type (**b**) and cluster ID (**c**). **d**, Stacked bar plots showing the abundance of each cluster in blood and tumors, respectively, from individuals with BrM (*n* = 6) and glioma (*n* = 3). **e**, Expression heat map showing the top ten genes in each CD8^+^ T cell cluster from *n* = 9 individuals. **f**, Enrichment of neoantigen-reactive CD8^+^ T cell gene signatures in each CD8^+^ T cell cluster, as indicated by the corresponding matched colors. The five different gene signatures analyzed are denoted by first author and year and can be found in the references. **g**, Stacked bar plot showing the clonality of the TCR in each CD8^+^ T cell cluster. TCR clonotypes are grouped into five categories based on their prevalence in each individual and are colored accordingly; ND, not detected. **h**, Alluvial plots visualizing the frequency of the top 15 TCR clonotypes within C1 (left) and C3 (right), respectively. Each clonotype within one cluster is annotated with a unique color. **i**, Number of TCR clonotypes perfectly matched with published virus-specific TCRs from VDJdb in each CD8^+^ T cell cluster from *n* = 9 individuals; CMV, cytomegalovirus; EBV, Epstein–Barr virus; HCV, hepatitis C virus. **j**, Abundance of C3 within each tumor. Samples are annotated as C3 high with a cumulative abundance of >30% (dotted horizontal line) or as C3 low. **k**, Dot plot showing average expression for each gene in *n* = 5,188 cells from C3 in C3-high tumors (*n* = 3 tumors) versus *n* = 920 cells from C3 in C3-low tumors (*n* = 6 tumors). Genes identified as DEGs in Seurat are highlighted. **l**, Bar plots showing the difference in abundance of defined T cell states in C3-high (*n* = 3 tumors) versus C3-low (*n* = 3 tumors) tumors depicted as fold change; Eff. Mem., effector memory; T_FH_, follicular helper T cells; T_H_1, type 1 helper T cells. Source data

**Extended Data Fig. 1:**
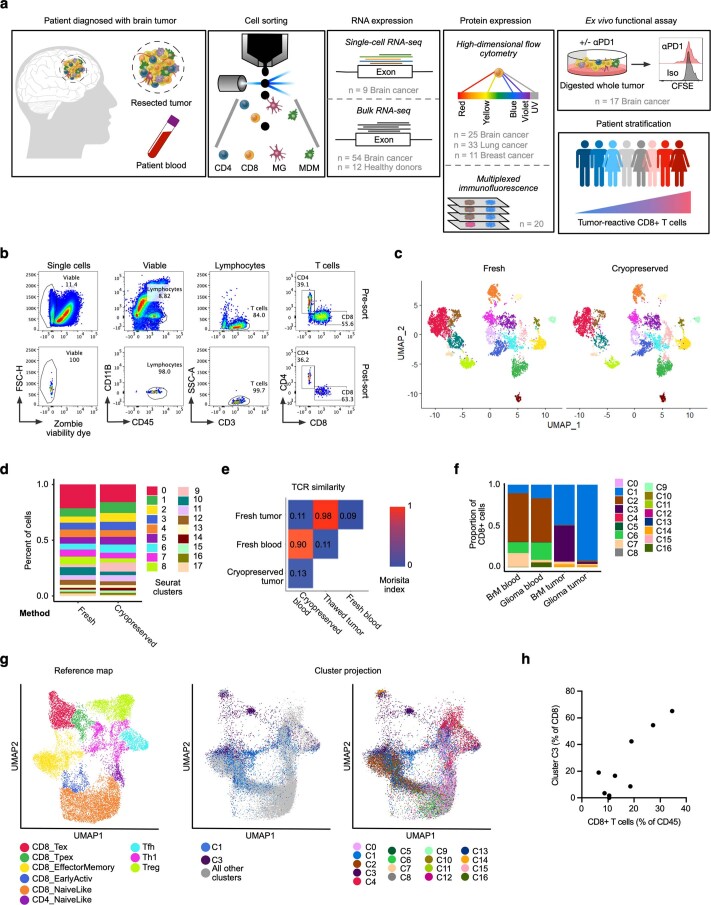
Single-cell RNAseq identifies pTRT in a subset of BrM patients and defines a BrM pTRT-specific gene signature. **a**, Schematic of the experimental analysis pipeline. **b**, Gating strategy for, and purity of, the sorting of T cells from whole tumor suspensions for single-cell RNAseq. **c**, Representative Uniform Manifold Approximation and Projection (UMAP) of blood and tumor T cells isolated from one patient, before and after cryopreservation. **d**, Stacked bar plots showing the frequency of each cluster before (fresh) and after (frozen) cryopreservation. **e**, Similarity analysis of the TCR repertoire in the blood and tumor of fresh and cryopreserved samples. **f**, Stacked bar plots showing the abundance of CD8 + clusters in BrM and glioma tumors and blood. **g**, Reference map of canonical T cell states (left panel) and projections (right panels) of T cells onto the map colored by cluster identity. **h**, Dot plot showing the relationship between the abundance of cluster C3 among CD8 + T cells and the frequencies of CD8 + T cells among all CD45 + immune cells in nine brain cancer patient samples. Source data

To assess the extent of phenotypical and functional heterogeneity of T cells in individuals with brain cancers, specifically gliomas and BrM, we used an integrated analytical pipeline^27^ to profile RNA and protein expression at the single-cell and bulk population levels (Extended Data Fig. 1a). We began by analyzing TILs and matched circulating T cells by single-cell RNA-seq (scRNA-seq) from six individuals with BrM and three individuals with glioma (Supplementary Table 1a and Extended Data Fig. 1b). Uniform manifold approximation and projection (UMAP) of 55,000 T cells in total showed substantial transcriptional differences between TILs and circulating T cells (Fig. 1a) and between CD4^+^ and CD8^+^ T cells (Fig. 1b). We identified 17 distinct clusters (Fig. 1c): seven CD8^+^ T cell clusters (C1, C2, C3, C6, C7, C14 and C16) and eight CD4^+^ T cell clusters (C0, C4, C5, C8, C9, C10, C11 and C13), with C4 representing regulatory T (T_reg_) cells (Fig. 1c and Supplementary Table 1b). C12 and C15 contained both CD4^+^ and CD8^+^ T cells. Cryopreservation did not alter the composition and phenotype of T cell subpopulations (Extended Data Fig. 1c–e), consistent with a previous study^28^.

Next, we compared cluster composition between individuals with glioma and BrM and found minimal variation in the blood (Fig. 1d and Extended Data Fig. 1f). By contrast, there were considerable differences in CD8^+^ TIL composition in gliomas with a high abundance of C1 and in BrM samples with a high abundance of C3 (Fig. 1d and Extended Data Fig. 1f). To determine cluster-specific genes for all CD8^+^ T cells, we performed differential expression analysis (DEA) and found that cells in C1 expressed *FTH1*, *CREM*, *GPR183*, *FOS* and *IL7R*, among other genes, but lacked expression of other activation markers, thereby suggesting an early activated state (Fig. 1e and Supplementary Table 1b)^29^. C3 was characterized by the expression of *CXCL13*, *RGS1*, *CTLA4*, *GZMB* and *IFNG* and multiple inhibitory receptors, indicating an overstimulated and dysfunctional phenotype (Fig. 1e and Supplementary Table 1b). Consequently, projection of our data onto a reference single-cell atlas of canonical T cell states^30^ revealed that C1 contained predominantly early active and effector memory CD8^+^ T cells, while cells in C3 primarily showed a precursor exhausted (T_pex_) or exhausted (T_ex_) phenotype (Extended Data Fig. 1g).

Because several studies have linked *CXCL13* expression and a dysfunctional state to tumor reactivity^16,22–26^, we hypothesized that C3 contains tumor-specific CD8^+^ T cells. We thus queried CD8^+^ T cells for expression of previously defined gene signatures of neoantigen-reactive CD8^+^ T cells^16,25,31–33^ (Supplementary Table 1c) and indeed found the highest expression in C3 (Fig. 1f). Moreover, 42% of T cells in C3 were substantially expanded (containing large and hyperexpanded clonotypes) versus 11% in C1, suggesting higher clonality in C3 (Fig. 1g). Additionally, tumor-expanded clonotypes in C3 were largely absent from the blood, indicating local expansion within the tumor, while those in C1 were also detected in the circulation, suggesting brain tumor-unrelated specificity, such as against viral antigens (Fig. 1h). Indeed, seven TCR clonotypes in C1 could be matched to published TCR sequences with validated antiviral specificities, while none were found among cells in C3 (Fig. 1i and Supplementary Table 1d). Together, these findings indicate that C3 contains pTRT cells.

We next analyzed C3 abundance in each individual and found substantial heterogeneity (Fig. 1j). All gliomas and half of the BrM samples had comparably low levels of these cells (<15% of CD8^+^ T cells) and were defined as C3 low, while the other half of BrM tumors were highly infiltrated with C3 cells (>30% of CD8^+^ T cells) and were classified as C3 high (Fig. 1j). The abundance of cells within C3, as determined by scRNA-seq, positively correlated with total CD8^+^ T cell frequencies acquired during cell sorting before single-cell encapsulation (Extended Data Fig. 1h). To investigate whether the phenotype of C3 cells also differed between these two groups, we performed DEA, which revealed that cells from C3-high tumors expressed higher levels of *TNFSF9*, *IFNG*, *GNLY*, *CCL3*, *CCL4* and *CCL4L2*, while those from C3-low samples showed higher expression of *FTH1*, *AREG* and *KLRB1*, among others. This suggested a more cytotoxic state of these cells in C3-high tumors (Fig. 1k).

Tumor-specific T cells can have different phenotypes, however, the majority show evidence of terminal exhaustion^31,33,34^. Comparison of canonical T cell states^30^ in C3-high versus C3-low tumors revealed substantially higher proportions of T_pex_ and T_ex_ cell gene signatures in C3-high tumors, while naive T cell gene signatures were more enriched in C3-low samples (Fig. 1l). Taken together, our scRNA-seq analysis identified a subset of BrM tumors infiltrated with pTRT cells.

### A subset of individuals with BrM show relatively high pTRT cell abundance

**Fig. 2:**
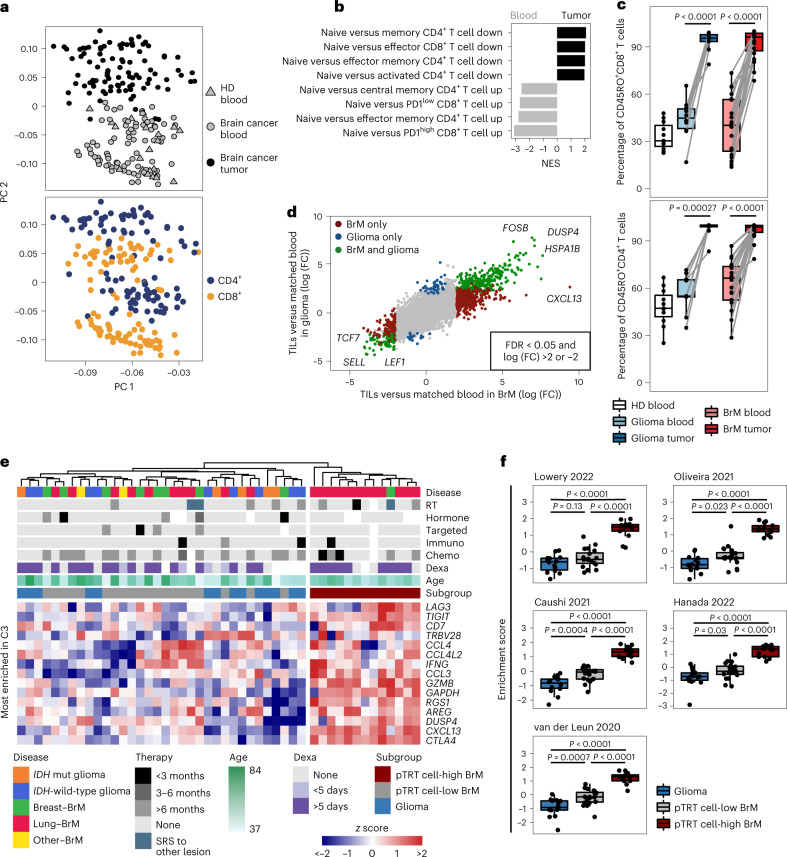
T cell activation and differentiation is a common feature of brain tumor-infiltrating T cells. **a**, Principal-component analysis based on the 250 most variably expressed genes (see Supplementary Table 2a for gene list) across all T cells (CD4^+^ and CD8^+^ from the blood and tumors) colored by tissue (top) or cell type (bottom) sorted from 54 individuals with brain cancer (tumor and blood) and blood from 12 HDs. For clinical details, see Supplementary Table 1a; PC, principal component. **b**, Bar plot showing GSEA of the comparison between T cells from blood (*n* = 85) and tumor (*n* = 102) samples using the MSigDB C7 collection filtered to contain only T cell-related pathways (see Supplementary Table 2b for full pathway list); NES, normalized enrichment score. **c**, Box plots representing the proportion of CD45RO^+^ cells among CD8^+^ (top) and CD4^+^ (bottom) T cells in *n* = 11 HD, *n* = 10 glioma and *n* = 19 BrM blood samples, and *n* = 9 glioma and *n* = 19 BrM tumor samples. Matched brain cancer samples are denoted by connected lines. Significance was determined by paired two-sided Wilcoxon test. Box plots represent first and third quartiles with the medians as the center; whiskers show 1.5× the interquartile range of the 25th and 75th percentiles. **d**, Dot plot showing the fold change (FC) of each gene between matched blood and tumor CD8^+^ T cells in *n* = 33 individuals with BrM (*x* axis) and *n* = 14 individuals with glioma (*y* axis). Genes passing the indicated significance cutoff in one or both diseases are colored as indicated. Most enriched shared genes in tumors or blood are highlighted, including *CXCL13* as the most DEG between glioma and BrM CD8^+^ TILs; FDR, false discovery rate. **e**, Expression heat map and hierarchical clustering of top genes from C3 in CD8^+^ T cells from *n* = 47 tumors in the bulk RNA-seq cohort. Columns and rows (*z* score) are hierarchically clustered. Disease and pTRT cell status are annotated per column; RT, radiotherapy; Hormone, hormone therapy; Targeted, targeted therapy; Immuno, immunotherapy; Chemo, chemotherapy; Dexa, dexamethasone; SRS, stereotactic radiosurgery. **f**, Box plots showing the enrichment of neoantigen-reactive CD8^+^ T cell gene signatures in CD8^+^ TILs in *n* = 14 individuals with glioma, *n* = 20 individuals with pTRT cell-low BrM and *n* = 13 individuals with pTRT cell-high BrM (five different signatures). Significance was determined with an unpaired two-sided Wilcoxon test and a Benjamini–Hochberg multiple comparison correction. Box plots are defined as in **c**. Source data

**Extended Data Fig. 2:**
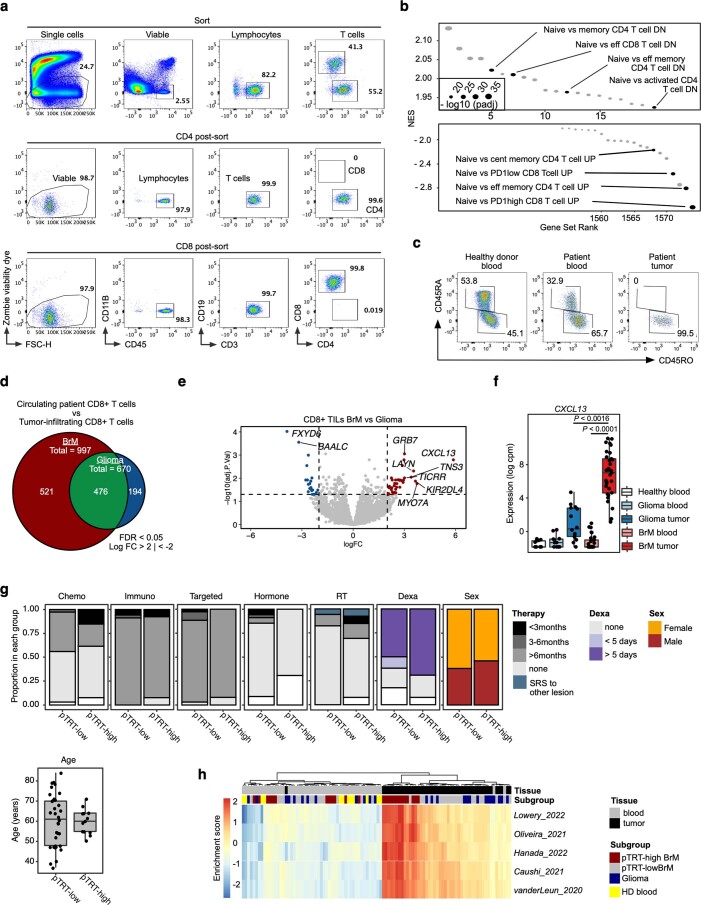
T cell activation and differentiation is a common feature of brain tumor-infiltrating T cells. **a**, Gating strategy to sort T cells from whole tumor for bulk RNA-seq and control of sorting purity. **b**, The most-enriched pathways in tumor or blood T cells from MSigDB C7 collection are shown. Pathways from (Fig. 2b) are highlighted. For full pathway list, see Supplementary Table 2b. **c**, Representative flow cytometry (FCM) plots showing the expression of CD45 isoforms in blood and tumor T cells. **d**, Venn diagram highlighting the private and shared differentially expressed genes (DEG) from the comparison of matched blood CD8 + T cells versus CD8 + tumor-infiltrating lymphocytes (TILs) using the indicated statistical cutoffs. **e**, Log2(fold-change) versus -log10(adjusted p value) volcano plot showing differential expression analysis contrasting CD8 + TILs from BrM to CD8 + TILs from glioma calculated with *limma* package in *R*. DEG with log2(fold-change) > 3 or log2(fold-change) < -3 and *P.adj* < 0.05 are highlighted. **f**, Box plots showing normalized RNA expression of *CXCL13* as log2(cpm) in CD8 + T cells from blood (n_healthy_ = 9, n_glioma_ = 9, n_BrM_ = 25), or tumor tissue (n_glioma_ = 14, n_BrM_ = 33). Adjusted *P*-values (Benjamini & Hochberg method) were calculated with *limma* package in *R*. **g**, Stacked bar plots visualizing clinical factors of the n = 47 patients grouped in Fig. 2f. No statistically significant differences between the pTRT-high and the pTRT-low group were observed with respect to patient treatment history, sex, or age (lower left panel). **h**, Heatmap of gene set variation analysis (GSVA) results of five neoantigen-reactive T cell gene signatures in CD8 + T cells isolated from tumors, matched patient blood, and healthy donor blood. Columns and rows are hierarchically clustered. For **f** and **g**, the box plots represent first and third quartiles with median as center; whiskers show 1.5 × interquartile range of the 25th and 75th percentiles. Source data

To expand our analyses beyond the 9 individuals in the scRNA-seq analysis, we next profiled a larger cohort, including TILs and matched blood T cells that were sorted from 54 individuals with brain cancer and the blood of 12 healthy donors (HDs), using population bulk RNA-seq (termed ‘bulk RNA-seq cohort’; Supplementary Table 1a and Extended Data Fig. 2a). Principal-component analysis revealed the largest differences between TILs and circulating T cells, while T cells from the blood of individuals with brain cancer or HDs clustered together (Fig. 2a, top, and Supplementary Table 2a). CD4^+^ and CD8^+^ T cell populations separated within both the blood and the tumor samples (Fig. 2a, bottom). Using the Molecular Signatures Database (MSigDB) C7 collection of immunologic signature gene sets, gene set enrichment analysis (GSEA) between blood T cells and TILs revealed CD4^+^ and CD8^+^ activation and differentiation gene sets among the highest enriched in TILs, while gene sets of naive T cells were enriched in the matched blood samples (Fig. 2b, Extended Data Fig. 2b and Supplementary Table 2b). To confirm these findings, we evaluated the expression of different CD45 isoforms by using FCM (Extended Data Fig. 2c). Compared to HDs and matched blood T cells, which were 40–50% CD45RO^+^, >90% of TILs were CD45RO^+^ (Fig. 2c). Taken together, these data indicate a conserved broad activation and differentiation program in brain TILs that appears to be independent of the underlying disease.

We next investigated the extent of inter-individual heterogeneity, particularly to uncover potential differences between glioma and BrM TILs when examined at higher granularity. We focused on CD8^+^ T cells (data are available for 47/54 individuals), as they can drive cytotoxic antitumor immunity and showed transcriptional differences between glioma and BrM in the scRNA-seq analysis (Fig. 1e). DEA between TILs and matched blood CD8^+^ T cells revealed 997 differentially expressed genes (DEGs) detected in BrM and 670 in glioma, of which, 476 were shared between both groups (Fig. 2d, Extended Data Fig. 2d and Supplementary Table 2c). Broad T cell activation markers, including *FOSB*, *DUSP4* and *HSPA1B*, were among the top shared DEGs expressed at higher levels in TILs (Fig. 2d). By contrast, genes indicative of a naive T cell state, such as *SELL*, *TCF7* and *LEF1*, were expressed at higher levels in circulating CD8^+^ T cells, thereby confirming the GSEA results (Fig. 2b,d). Direct comparison of BrM CD8^+^ TILs with glioma CD8^+^ TILs revealed *CXCL13* as the most highly DEG (Fig. 2d and Extended Data Fig. 2e). Of note, the expression of *CXCL13* in BrM TILs showed substantial inter-individual heterogeneity (Extended Data Fig. 2f).

As *CXCL13* was also the most enriched gene in the scRNA-seq pTRT cell cluster C3, we next analyzed CD8^+^ TILs for expression of the most significantly enriched genes in C3 (fold change > 1; Supplementary Table 1b). Hierarchical clustering separated the bulk RNA-seq cohort with brain cancer into two major groups: (1) mostly lung–BrM samples showing high expression of C3-specific genes and (2) gliomas and all other BrM samples with relatively low expression (Fig. 2e). Importantly, this separation of samples was independent of individual age (*P* = 0.51, Welch’s *t*-test), prior therapy (*P* = 0.75, Fisher’s exact test) or dexamethasone treatment (*P* > 0.99, Fisher’s exact test; Extended Data Fig. 2g). We hypothesized that tumors with elevated expression of C3-specific genes were infiltrated with a relatively high proportion of tumor-reactive CD8^+^ T cells and thus labeled them as pTRT cell-high BrM (Fig. 2e). The other BrM samples and all gliomas were designated pTRT cell-low, and we maintained this distinction throughout the study. In line with this reasoning, CD8^+^ TILs from pTRT cell-high BrM showed a significantly higher enrichment of multiple neoantigen-reactive CD8^+^ T cell gene signatures than pTRT cell-low BrM or glioma (Fig. 2f). By contrast, no enrichment of these gene signatures was observed in circulating CD8^+^ T cells from the three subgroups (Extended Data Fig. 2h).

### Abundant and clonally expanded CD8^+^ T cells in pTRT cell-high BrM

**Fig. 3:**
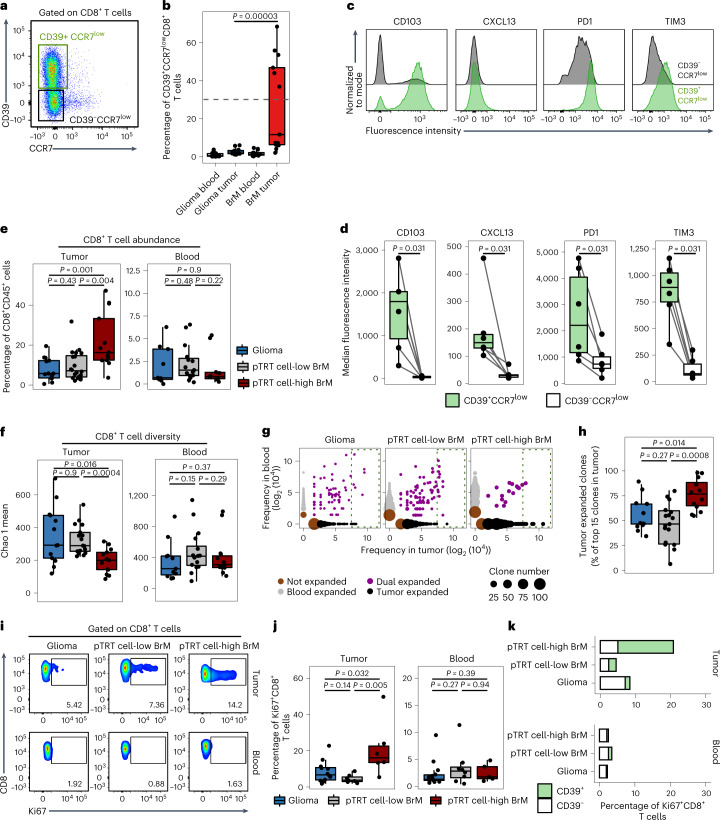
CD8^+^ T cells in pTRT cell-high tumors are highly abundant and clonally expanded. **a**, Representative FCM plot showing the gating strategy for CD39^+^CCR7^low^CD8^+^ and CD39^–^CCR7^low^CD8^+^ T cells. **b**, Box plot showing the percentage of CD39^+^CCR7^low^ cells among CD8^+^ T cells in individuals with glioma (*n* = 14 blood and *n* = 12 tumor samples) and BrM (*n* = 13 blood and *n* = 13 tumor samples). Significance was determined with a two-sided Wilcoxon test and Benjamini–Hochberg multiple comparison correction. **c**, FCM histograms showing the expression of indicated markers in one representative pTRT cell-high sample from an individual with BrM. **d**, Box plots comparing the median fluorescence intensity of each of the markers indicated in CD39^+^CCR7^low^CD8^+^ and CD39^–^CCR7^low^CD8^+^ T cells in *n* = 6 individuals with pTRT cell-high BrM. Significance was determined with a paired two-sided Wilcoxon test. **e**, Box plots showing the abundance of CD8^+^ T cells in tumors (left, *n* = 14 glioma, *n* = 20 pTRT cell-low and *n* = 13 pTRT cell-high BrM samples) and blood (right, *n* = 9 glioma, *n* = 15 pTRT cell-low and *n* = 10 pTRT cell-high BrM samples) as the proportion of all CD45^+^ immune cells. Significance was determined by unpaired two-sided Wilcoxon test with a Benjamini–Hochberg multiple comparison correction. **f**, Box plots summarizing the diversity of TCR β-chain in tumors (left, *n* = 13 glioma, *n* = 17 pTRT cell-low and *n* = 13 pTRT cell-high BrM samples) and blood (right, *n* = 9 glioma, *n* = 15 pTRT cell-low and *n* = 11 pTRT cell-high BrM samples) as the Chao 1 index. Significance was determined by unpaired two-sided Wilcoxon test with a Benjamini–Hochberg multiple comparison correction. **g**, Scatter plots of TCR clones are shown for three representative individuals with normalized frequency of clones in the tumor and blood. Dots are colored by expansion profile (not expanded, expanded only in tumor, expanded only in blood or dually expanded) and sized by the number of clones with the same expansion statistic. **h**, Box plot summarizing the proportion of clones detected only in the tumor from the 15 most-expanded clones in *n* = 11 individuals with glioma, *n* = 17 individuals with pTRT cell-low BrM and *n* = 12 individuals with pTRT cell-high BrM. Significance was determined by unpaired two-sided Wilcoxon test with a Benjamini–Hochberg multiple comparison correction. **i**, Representative FCM plots showing Ki67 staining gated on CD8^+^ T cells. **j**, Box plots showing the percentage of Ki67^+^ cells among CD8^+^ T cells in *n* = 12 glioma, *n* = 7 pTRT cell-low BrM and *n* = 6 pTRT cell-high BrM tumor samples, and *n* = 14 glioma, *n* = 8 pTRT cell-low BrM and *n* = 5 pTRT cell-high BrM blood samples. Significance was determined by unpaired two-sided Wilcoxon test with a Benjamini–Hochberg multiple comparison correction. **k**, Stacked bar plots showing the mean proportion of Ki67^+^CD8^+^ T cells in *n* = 12 glioma, *n* = 7 pTRT cell-low BrM and *n* = 6 pTRT cell-high BrM tumor samples, and *n* = 14 glioma, *n* = 8 pTRT cell-low BrM and *n* = 5 pTRT cell-high BrM blood samples. CD39 positivity is indicated in green. Box plots in **b**, **d**–**f**, **h** and **j** are defined as explained in Fig. 2c. Source data

**Extended Data Fig. 3:**
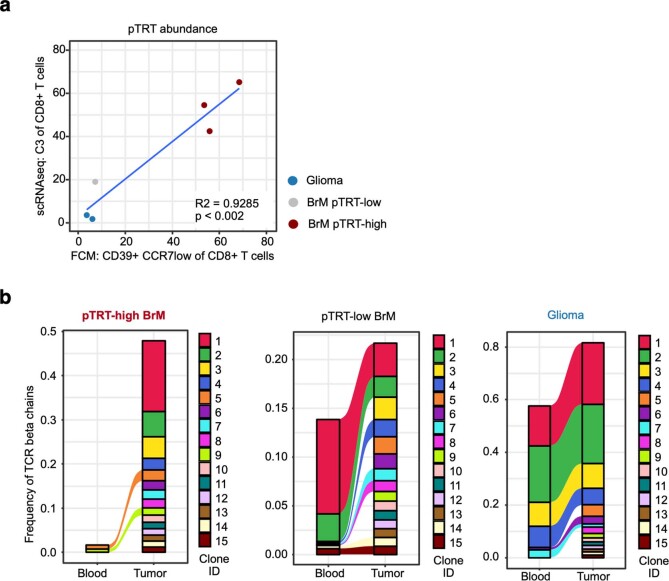
CD8 + pTRT are expanded predominantly in tumor tissue and can be detected by flow cytometry as CD39 + CCR7low cells. **a**, Dot plot showing the relationship between the abundance of cluster C3 among CD8 + T cells and the frequencies of CD39 + CCR7low cells among all CD8 + T cells in six brain tumors profiled by FCM and scRNA-seq. Significance determined with linear regression. **b**, CD8 + T cells in pTRT-high tumors are highly abundant and clonally expanded. Representative alluvial plots visualizing the frequency of the top 15 T cell receptor (TCR) clones within three patients (pTRT-high BrM, pTRT-low BrM, and a glioma patient) in the blood and tumor, respectively. Each clone within an individual patient sample is annotated with a unique color. Source data

To validate the transcriptome-based findings with an independent method and an independent cohort, we next analyzed 25 brain tumor samples by FCM (3/25 were also profiled by RNA-seq). We used CD39 as a marker of putative tumor-reactive CD8^+^ T cells, as recently proposed in different types of cancer including non-small cell lung cancer (NSCLC), head and neck squamous cell carcinoma, colorectal cancer and melanoma^31,33,35,36^ (Fig. 3a). Cells were further gated as CCR7^low^ to eliminate potential contamination by bystander naive and memory cells^34^. CD8^+^CD39^+^CCR7^low^ cells were almost undetectable in the blood of individuals with BrM and glioma, while among TILs, we found a significantly higher proportion in BrM than in glioma (Fig. 3b). Similar to the transcriptome analysis, this difference was driven by a subgroup of individuals with BrM (6/13) with high abundance of CD39^+^CCR7^low^ TILs (>30% of all CD8^+^ T cells). We observed a significant correlation (*R*^2^ = 0.9285, *P* < 0.002) between the proportion of CD39^+^CCR7^low^ TILs detected by FCM and the abundance of C3 in the scRNA-seq data (Extended Data Fig. 3a). Within individual samples showing high accumulation of CD39^+^CCR7^low^ TILs, we compared the expression of four molecules related to neoantigen-reactive T cells on CD39^+^CCR7^low^ versus CD39^–^CCR7^low^ cells (Fig. 3c). CD39^+^CCR7^low^ cells expressed CD103 (encoded by *ITGAE*), CXCL13, PD1 (encoded by *PDCD1*) and TIM3 (encoded by *HAVCR2*) at significantly higher levels than their CD39^–^ counterparts, indicating that the CD39^+^CCR7^low^ population indeed contains pTRT cells (Fig. 3d).

RNA-seq and FCM analyses independently demonstrated heterogeneous abundance of potentially tumor-specific CD8^+^ T cells in brain tumors, enabling the separation of the cohorts into three groups: glioma (pTRT cell-low), pTRT cell-low BrM and pTRT cell-high BrM. Comparison of these groups revealed substantially higher proportions of total CD8^+^ TILs among all tumor-infiltrating immune cells in pTRT cell-high BrM than in pTRT cell-low BrM or gliomas (Fig. 3e, left). No significant differences in CD8^+^ T cell proportions were observed in the blood (Fig. 3e, right). Therefore, we investigated whether the increased proportion of CD8^+^ TILs could be a consequence of clonal expansion and analyzed the diversity of the TCR β-chain. We found that CD8^+^ TILs, but not circulating cells, from pTRT cell-high BrM had a significantly lower TCR diversity than pTRT cell-low BrM or gliomas, thus suggesting a larger clonal expansion in these samples (Fig. 3f). Furthermore, a substantial proportion of highly expanded TCRs in pTRT cell-high BrM were not detected in matched blood samples, potentially indicating tumor-specific expansion (Fig. 3g,h and Extended Data Fig. 3b). By contrast, expanded TCRs in pTRT cell-low BrM and gliomas were also frequently present in the blood, suggesting a cancer-unrelated specificity, such as against viral antigens (Fig. 3g,h and Extended Data Fig. 3). Moreover, FCM analysis revealed more Ki67^+^CD8^+^ T cells in pTRT cell-high BrM than in other brain tumors (Fig. 3i,j). Of note, the majority of Ki67^+^ cells in pTRT cell-high BrM were CD39^+^ (Fig. 3k). Together, these findings further support the hypothesis that pTRT cell-high BrM harbor a relatively high proportion of clonally expanded tumor antigen-specific CD8^+^ T cells.

### pTRT cells are found in BrM perivascular niches (PVNs), stroma and tumor nests

**Fig. 4:**
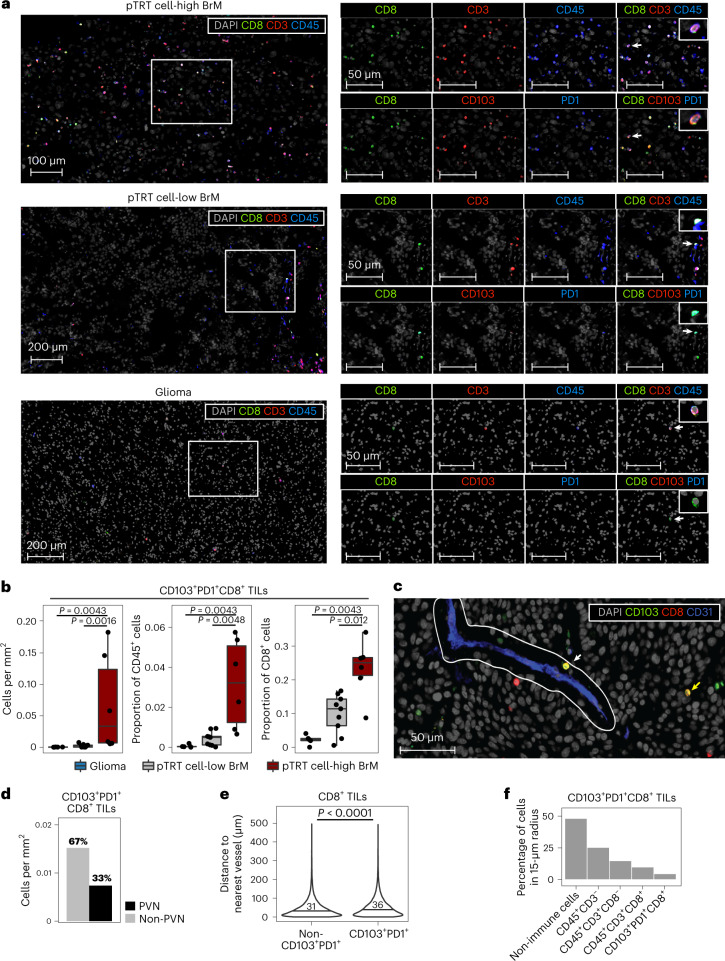
pTRT cells in BrM are located in PVNs and the stroma and within tumor nests. **a**, Representative IF images from a pTRT cell-high BrM, a pTRT cell-low BrM and a glioma. Insets on the far right of each image show a higher magnification of the CD8^+^ T cell indicated by a white arrow. **b**, Box plots showing the quantification of CD103^+^PD1^+^CD8^+^ TILs in *n* = 5 glioma, *n* = 9 pTRT cell-low BrM and *n* = 6 pTRT cell-high BrM tumor samples, shown as cells per square millimeter (left), proportion of CD45^+^ cells (middle) and proportion of CD8^+^ cells (right). Significance was determined by unpaired two-sided Wilcoxon test with a Benjamini–Hochberg multiple comparison correction. Box plots are defined as explained in Fig. 2c. **c**, Representative image of T cells within (white arrow) and outside (yellow arrow) the PVN, defined as a 15-µm radius surrounding the nearest vessel. **d**, Quantification of CD103^+^PD1^+^CD8^+^ TILs within or outside the PVN (*n* = 79,586 CD8^+^ T cells). **e**, Violin plot showing the mean distance of CD8^+^ TILs from the respective nearest vessel stratified by the coexpression of CD103 and PD1. The mean distance for each group is indicated. Significance was determined by unpaired two-sided Wilcoxon test. **f**, Neighborhood analysis summarizing the cell types within a 20-µm radius around CD103^+^PD1^+^CD8^+^ TILs. Source data

**Extended Data Fig. 4:**
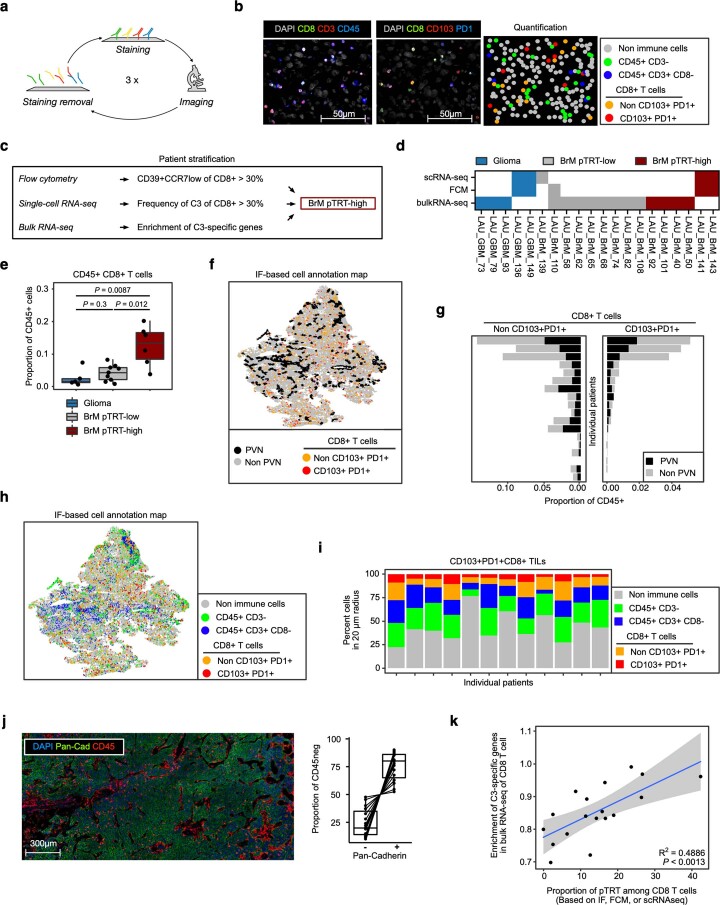
pTRT in BrM are located in perivascular niches, stroma, and within tumor nests. **a**, Schematic of multiplexed immunofluorescence (IF) imaging. **b**, Representative IF images and quantification using QuPath and R software from one patient are shown. **c**, Patient stratification strategy for IF analysis based on FCM, scRNA-seq, and bulk RNA-seq data. **d**, Heatmap summarizing the pTRT status for each patient using the strategy described in (c). **e**, Box plot showing the abundance of CD8 + T cells in tumors (n_glioma_ = 5, n_BrM_pTRT-low_ = 9, and n_BrM_pTRT-high_ = 6) as the proportion of all CD45 + immune cells detected by IF. Significance determined with unpaired two-sided Wilcoxon-test with Benjamini-Hochberg multiple comparison correction. **f**, Full section with IF-derived cell annotation of the indicated populations and tissue regions for one representative patient sample. **g**, Stacked bar plots showing the quantification of the location of CD103 + PD1 + CD8 + TILs and all other CD8 + TILs in each individual patient. Color indicates the location within (perivascular niches, PVN) or outside (non-PVN) a 15 µm radius surrounding the nearest vessel. **h**, Full section IF-derived annotation of the indicated cell populations for one representative patient sample. **i**, Neighborhood analysis summarizing the cell types within a 20 µm radius around CD103 + PD1 + CD8 + TILs in individual patients with > ten CD103 + PD1 + CD8 + cells in each analyzed tissue section. **j**, Representative image (left panel) and quantification (right panel) of the proportion of tumor cells (pan-cadherin + ) among all CD45neg non-immune cells in n = 19 brain metastasis samples. **k**, Dot plot showing the relationship between the enrichment of C3-specific genes in CD8 + T cells profiled by bulk RNA-seq and the frequencies of pTRT measured by IF, FCM, and scRNA-seq. Significance determined with linear regression. Confidence interval of 0.95 is indicated in grey. For **e** and **j**, the box plots represent first and third quartiles with median as center; whiskers show 1.5 × interquartile range of the 25th and 75th percentiles. Source data

We next queried the spatial organization of T cells, and especially pTRT cells, in brain tumors. We performed multiplexed IF staining of tissue sections from 20 individuals with brain cancer (Extended Data Fig. 4a). Because CXCL13 (secreted protein) and CD39 (expressed at higher levels on vessels than on T cells) were not suitable for IF, we used the coexpression of CD103 and PD1 on CD45^+^CD3^+^CD8^+^ T cells as a readout for potential tumor specificity (Extended Data Fig. 4b). Individuals with BrM were stratified into pTRT cell-high or pTRT cell-low, respectively, based on the frequency of C3 determined by scRNA-seq, the enrichment of C3-specific genes in CD8^+^ T cells analyzed by bulk RNA-seq or the abundance of CD39^+^CCR7^low^CD8^+^ T cells detected by FCM (Extended Data Fig. 4c). Group assignment by the different methods was identical in those samples analyzed by more than one technology (Extended Data Fig. 4d). CD103^+^PD1^+^CD8^+^ TILs were substantially more abundant in pTRT cell-high BrM than in pTRT cell-low BrM or gliomas (Fig. 4a,b). Moreover, we observed higher total CD8^+^ TIL infiltration in pTRT cell-high BrM by IF (Extended Data Fig. 4e), recapitulating the FCM analysis in Fig. 3e.

Because blood vessels are the main entry site for T cells into brain tumors, we next studied the spatial relationship between the vasculature and CD103^+^PD1^+^CD8^+^ TILs. We defined the PVN as a region surrounding CD31^+^ vessels with a diameter of 15 µm and found that one-third of CD103^+^PD1^+^CD8^+^ TILs were located within the PVN (Fig. 4c,d and Extended Data Fig. 4f,g). Two-thirds of pTRT cells were located outside the PVN and were infiltrating the brain tumor tissue (Fig. 4d and Extended Data Fig. 4f,g). CD103^+^PD1^+^CD8^+^ TILs were significantly further away from the nearest vessel than all other CD8^+^ TIL populations (denoted ‘non-CD103^+^PD1^+^’), indicating deeper penetration into the tissue (Fig. 4e). Neighborhood analysis showed that the majority of CD103^+^PD1^+^CD8^+^ TILs were in close proximity to non-immune cells (Fig. 4f and Extended Data Fig. 4h,i). A separate analysis of the same tissues revealed that, on average, 80% of CD45^–^ non-immune cells in BrM were pan-cadherin^+^ tumor cells, indicating that pTRT cells are present within tumor nests (Fig. 4f and Extended Data Fig. 4j).

Finally, we combined the results from the single-cell methods (scRNA-seq, FCM and IF) to validate the observations generated by bulk RNA-seq of sorted CD8^+^ T cells (Fig. 2). We found a significant positive correlation between the true abundance of pTRT cells among all CD8^+^ T cells (measured by scRNA-seq, FCM and IF) and the computationally imputed pTRT cell frequencies in bulk RNA-seq data using C3-specific genes (Extended Data Fig. 4k), confirming the validity of this approach.

### A distinct myeloid cell subtype is associated with high pTRT cell abundance

**Fig. 5:**
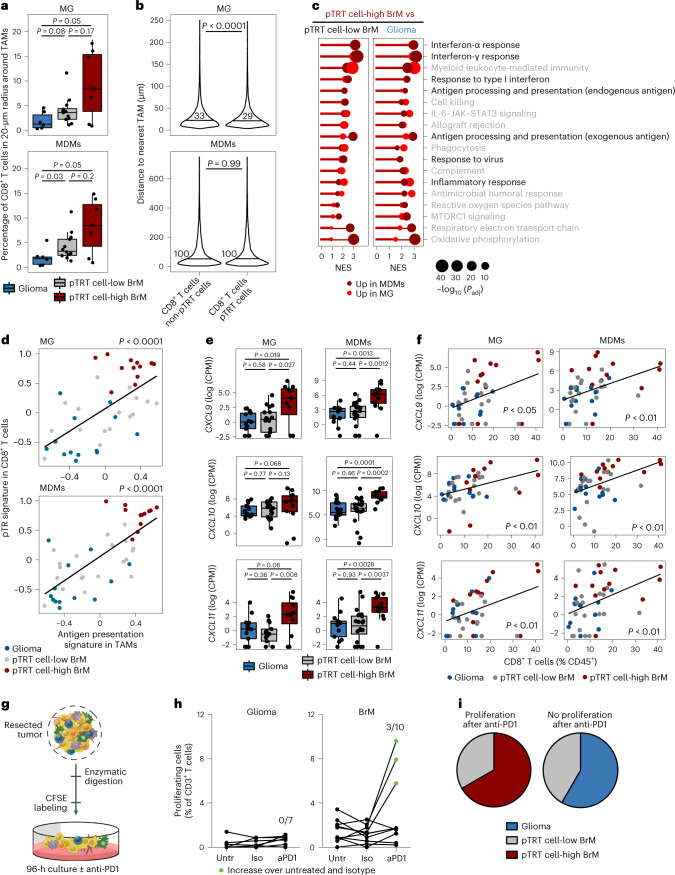
Myeloid cells with antigen presentation capacity are associated with high pTRT cell abundance. **a**, Box plots showing the percentage of CD8^+^ TILs within a 20-µm radius around MG (top) or MDMs (bottom) in *n* = 6 individuals with glioma, *n* = 12 individuals with pTRT cell-low BrM and *n* = 7 individuals with pTRT cell-high BrM. Significance was determined by unpaired two-sided Wilcoxon test with a Benjamini–Hochberg multiple comparison correction. **b**, Violin plots showing the distance of CD8^+^ TILs from the respective nearest TAM (MG, top; MDMs, bottom) stratified by the coexpression of CD103 and PD1 in *n* = 5 individuals with glioma and *n* = 16 individuals with BrM. The mean distance for each group is indicated. Significance was determined by unpaired two-sided Wilcoxon test. **c**, Balloon plot showing results from a GSEA of Hallmark and Gene Ontology biological process gene sets in MDMs and MG from pTRT cell-high BrM versus pTRT cell-low BrM or gliomas, respectively. Pathways mentioned in the main text are highlighted in black. Adjusted *P* values (Benjamini–Hochberg method) and normalized enrichment scores (NES) were calculated with the fgsea package in R; IL-6, interleukin 6; *P*_adj_, adjusted *P* value. **d**, Dot plots indicating a correlation between the expression of antigen presentation programs in MG (top) or MDMs (bottom) and the pTRT cell signature enrichment in CD8^+^ T cells. Significance was determined by linear regression. Dots are colored by disease group; *n* = 13 individuals with glioma, *n*_MG_ = 17 individuals with pTRT cell-low BrM, *n*_MDM_ = 20 individuals with pTRT cell-low BrM and *n* = 12 individuals with pTRT cell-high BrM. **e**, Box plots showing the expression of T cell recruitment cytokines as log_2_ (CPM) in *n* = 13 individuals with glioma, *n*_MG_ = 17 individuals with pTRT cell-low BrM, *n*_MDM_ = 20 individuals with pTRT cell-low BrM and *n* = 12 individuals with pTRT cell-high BrM. Adjusted *P* values (Benjamini–Hochberg method) were calculated with the limma package in R. CPM, counts per million. **f**, Dot plots indicating the correlation between the abundance of CD8^+^ T cells and the expression of *CXCL9*, *CXCL10* and *CXCL11* in MG (left) or MDMs (right). Significance was determined by linear regression. Dots are colored by disease group; *n* = 13 individuals with glioma, *n*_MG_ = 17 individuals with pTRT cell-low BrM, *n*_MDM_ = 20 individuals with pTRT cell-low BrM and *n* = 12 individuals with pTRT cell-high BrM. **g**, Schematic of the ex vivo anti-PD1 treatment and T cell proliferation assay. **h**, Summary of ex vivo T cell proliferation in *n* = 7 glioma (left) and *n* = 10 BrM (right) tumor samples under the indicated conditions. Samples showing a proliferation increase compared to both untreated (Untr) and isotype control (Iso) conditions are highlighted; aPD1, anti-PD1. **i**, Pie charts indicating the disease group of *n* = 7 glioma and *n* = 8 BrM tumors used in the ex vivo proliferation assay profiled by at least one other method (FCM or RNA-seq) and grouped by their response to anti-PD1. Box plots in Fig. 5a,e are defined as explained in Fig. 2c. Source data

**Extended Data Fig. 5:**
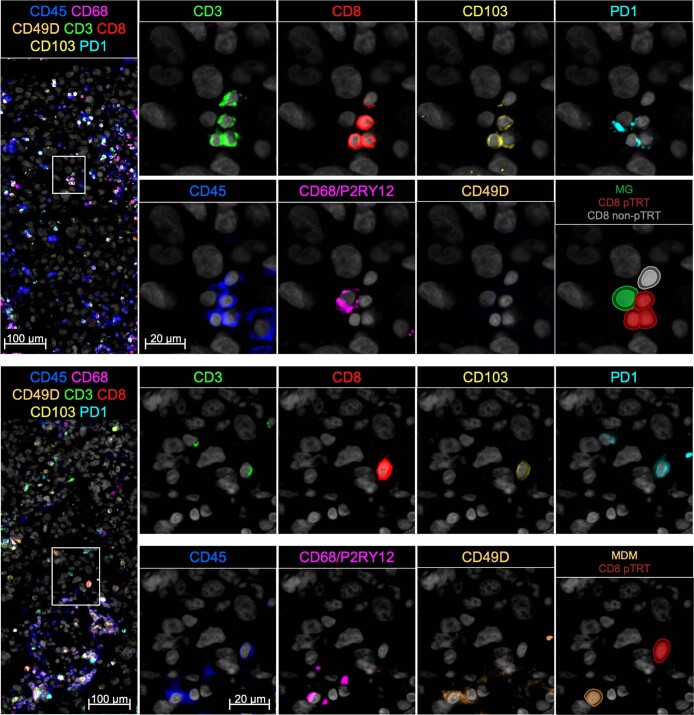
Distance of myeloid cells to CD8 + TILs. Representative IF images and quantification using QuPath and R software from two patients are shown, indicating T cells, MG (upper set of images) and MDMs (lower set of images).

We next investigated whether high pTRT cell abundance was associated with a distinct tumor microenvironment, particularly with respect to myeloid cells, which have been shown to regulate the function of tumor-specific T cells in kidney, prostate, bladder and ovarian cancer^37,38^. Spatial imaging analysis of >4 × 10^6^ cells across 25 samples revealed that in pTRT cell-high BrM, a larger proportion of CD8^+^ TILs was in close proximity (20 μm) to tumor-associated macrophages (TAMs), which include resident microglia (MG; identified as CD45^+^CD68^+^ and/or P2RY12^+^CD49D^–^) and recruited monocyte-derived macrophages (MDMs; identified as CD45^+^CD68^+^ and/or P2RY12^+^CD49D^+^), compared to glioma or pTRT cell-low BrM (Fig. 5a), allowing for potential direct cellular interactions. This observation could be partially influenced by the higher proportion of total CD8^+^ TILs in pTRT cell-high BrM (Extended Data Fig. 4e). Thus, we next analyzed the distance between CD8^+^ TILs and their respective nearest TAMs. We found that CD103^+^PD1^+^CD8^+^ TILs were located significantly, albeit modestly, closer to the nearest MG and had the same distance to the nearest MDM as all other CD8^+^ TILs (Fig. 5b and Extended Data Fig. 5). These results indicate a similar likelihood for pTRT cells and non-pTRT cells to communicate with TAMs.

**Extended Data Fig. 6:**
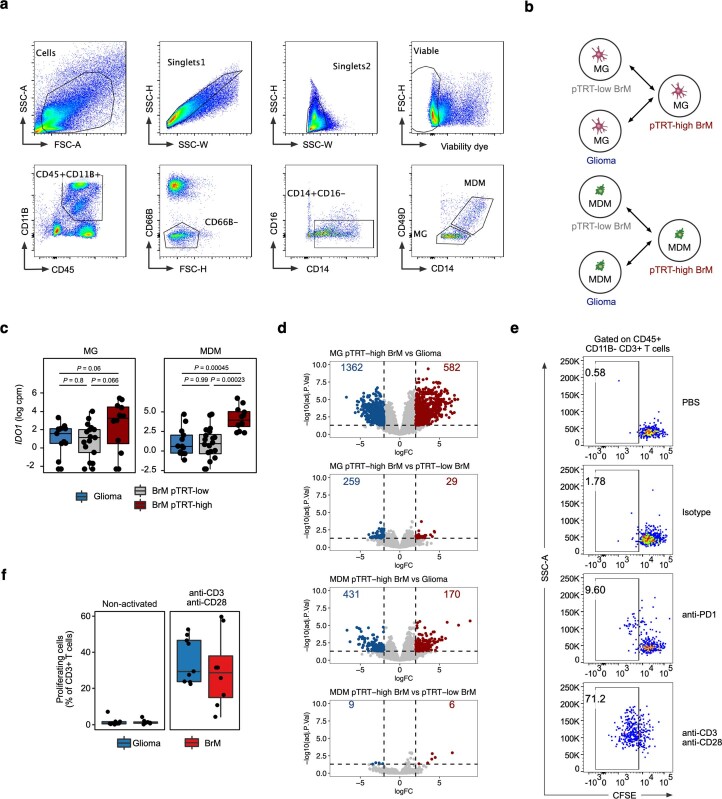
Myeloid cells with antigen-presentation capacity are associated with high pTRT abundance. **a**, Gating strategy to sort MG and MDM from whole tumors for bulk RNA-seq. **b**, Schematic of the differential expression analysis (DEA). **c**, Box plots showing expression of *IDO1* as log2(cpm) in n_MG_ = 42 and n_MDM_ = 45 patients. Adjusted *P*-values (Benjamini & Hochberg method) were calculated with *limma* package in *R*. **d**, Log2(fold-change) versus -log10(adjusted *P*-value, Benjamini & Hochberg method) volcano plot showing DEA results contrasting MG or MDMs from pTRT-high BrM to respective cells in pTRT-low BrM or glioma tumors calculated with *limma* package in *R*. Number of DEG using log2(fold-change) > 2 or log2(fold-change) < -2 and *P.adj* < 0.05 as cutoff are indicated. For full gene lists, see Supplementary Table 3b. **e**, Representative FCM plots showing CFSE dilution in CD3 + cells from one tumor sample under the experimental conditions indicated. Percentage of proliferating T cells is annotated for each panel. **f**, Box plots summarizing the proliferation of T cells isolated from n = 9 glioma or n = 8 BrM tumors, respectively, with or without anti-CD3/anti-CD28 stimulation. For **c** and **f**, the box plots represent first and third quartiles with median as center; whiskers show 1.5 × interquartile range of the 25th and 75th percentiles. Source data

We next examined whether there was a difference in the potential for TAM–T cell communication specifically when comparing the pTRT cell-high and cell-low tumors identified herein. We analyzed the transcriptomes of sorted MG and MDMs from the same tumor samples from which we had also collected T cells (Extended Data Fig. 6a,b). GSEA using Hallmark and Gene Ontology biological process gene sets revealed substantial differences in both MG and MDMs (Fig. 5c and Supplementary Table 3a). In particular, gene sets associated with interferon response and antigen presentation were highly enriched in TAM populations isolated from pTRT cell-high BrM compared to those in pTRT cell-low BrM and glioma samples (Fig. 5c). Moreover, there was a robust correlation between the enrichment of antigen presentation genes in TAMs and the enrichment of pTRT cell signature genes in CD8^+^ T cells (Fig. 5d). DEA confirmed the elevation of interferon response and antigen presentation genes and also revealed significantly higher expression of the T cell recruitment molecules *CXCL9*, *CXCL10* and *CXCL11* in TAMs from pTRT cell-high BrM (Fig. 5e, Extended Data Fig. 6b and Supplementary Table 3b). Furthermore, the expression level of T cell-recruiting chemokines in TAMs correlated significantly with the abundance of CD8^+^ T cells in the same samples (Fig. 5f). Of note, in addition to the ability to recruit and activate T cells, TAMs from pTRT cell-high BrM expressed high levels of *IDO1*, suggesting that they may also possess T cell-suppressive properties (Extended Data Fig. 6c).

Myeloid antigen-presenting cell niches have also been shown to regulate anti-PD1 efficacy^37^, and *CXCL9* expression in the tumor microenvironment can predict response to ICB therapy^23^. We therefore next evaluated whether brain TIL proliferation could be stimulated with anti-PD1 treatment ex vivo. We used a previously established 96-h experimental assay^37^ and measured carboxyfluoroscein succinimidyl ester (CFSE) dilution specifically in T cells by FCM (Fig. 5g). It is important to note that we were unable to incorporate cognate tumor antigen peptides in this experimental design due to the current lack of validated common targets in primary and metastatic brain cancers. Nonetheless, using this strategy, we found that T cell proliferation could not be increased ex vivo via anti-PD1 treatment in glioma samples (zero of seven), while in BrM, we observed a proliferation increase following anti-PD1 treatment in three of ten samples (Fig. 5h and Extended Data Fig. 6d). By contrast, nonspecific activation with anti-CD3/anti-CD28 induced proliferation in glioma and BrM TILs to a similar extent, suggesting that functional T cells can be found in both pathologies (Extended Data Fig. 6e,f). Fifteen of the 17 tumors analyzed for proliferation following anti-PD1 treatment in this assay were also profiled by bulk RNA-seq, FCM or IF and thus contained information regarding pTRT cell status. Two of three proliferating samples were pTRT cell-high BrM, while non-proliferating samples contained only pTRT cell-low BrM and gliomas (Fig. 5i). Thus, interferon-stimulated TAMs with antigen-presenting and T cell-recruiting capacities in certain BrM lesions may represent an important mechanism that enables the high abundance of pTRT cells in vivo and that may potentially influence the response to anti-PD1 treatment.

### Comparison of pTRT cells in BrM versus extracranial cancer

**Fig. 6:**
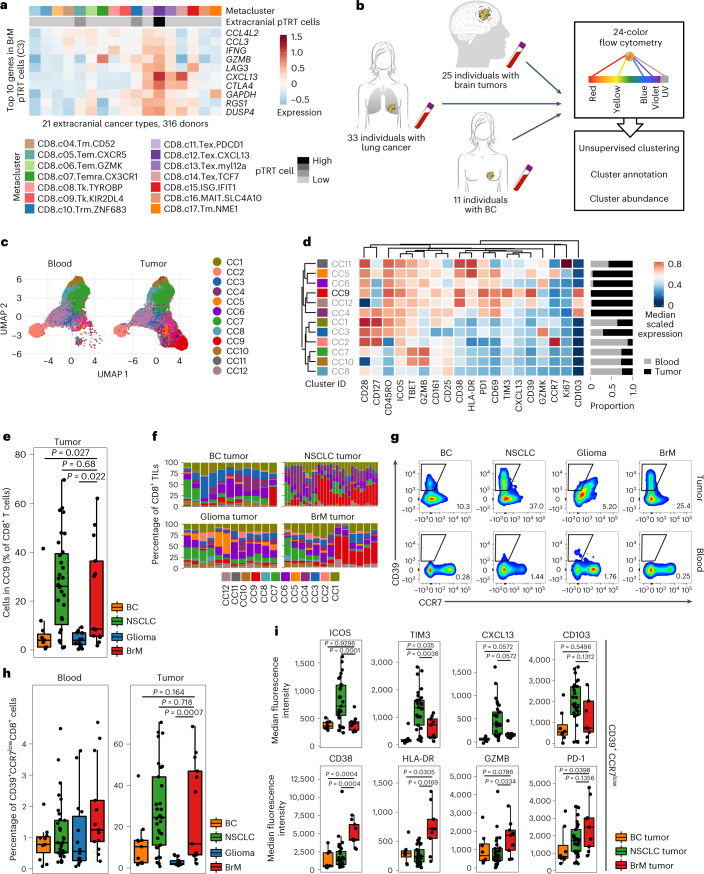
Abundance of pTRT cells in BrM and primary NSCLC is comparable, while the phenotypes are distinct. **a**, Expression heat map of the top ten genes from BrM C3 in the pan-cancer single-cell T cell atlas data. Rows represent normalized and scaled expression. Metacluster and pTRT cell status are annotated per column. **b**, Schematic of the high-dimensional FCM analysis. **c**, UMAP of the full cohort grouped by tissue with the cytometry cluster (CC) annotated. **d**, Heat map showing median scaled expression of individual markers in each cluster and the proportion among blood or tumor CD8^+^ T cells. **e**, Box plot visualizing the abundance of the pTRT cell cluster CC9 in *n* = 11 individuals with BC, *n* = 32 individuals with NSCLC, *n* = 12 individuals with glioma and *n* = 12 individuals with BrM. Significance was determined with a Kruskal–Wallis test and a Benjamini–Hochberg multiple comparison correction. **f**, Stacked bar plots showing the abundance of each cluster in individual tumors grouped by disease. **g**, Representative FCM plots illustrating the expression of CD39 and CCR7 in the blood and tumor of each disease group. **h**, Box plot summarizing the abundance of CD39^+^CCR7^low^ cells among all CD8^+^ T cells in the blood of *n* = 11 individuals with BC, *n* = 33 individuals with NSCLC, *n* = 14 individuals with glioma and *n* = 13 individuals with BrM, and in the tumors of *n* = 11 individuals with BC, *n* = 32 individuals with NSCLC, *n* = 12 individuals with glioma and *n* = 13 individuals with BrM. Significance was determined with a Kruskal–Wallis test with a Benjamini–Hochberg multiple comparison correction. **i**, Box plots showing median fluorescence intensity of the indicated markers expressed on CD39^+^CCR7^low^CD8^+^ TILs from *n* = 8 individuals with BC, *n* = 29 individuals with NSCLC and *n* = 9 individuals with BrM. Significance was determined by a Kruskal–Wallis test with a Benjamini–Hochberg multiple comparison correction. Box plots in Fig. 6e,h,i are defined as explained in Fig. 2c. Source data

While little is currently known about pTRT cells in brain cancers, these cells have been investigated in several extracranial tumor types^26^. Therefore, we next compared pTRT cells in intracranial and extracranial tumors. We began by analyzing scRNA-seq data from 21 different extracranial cancer types and 316 individuals^26^ for the expression of the top ten genes in BrM pTRT cell scRNA-seq cluster C3. Interestingly, we found the highest expression of all ten genes within the CD8.c12.Tex.CXCL13 cluster, which has been annotated to contain the largest proportion of pTRT cells, thereby indicating similarities between intracranial and extracranial pTRT cells (Fig. 6a).

**Extended Data Fig. 7:**
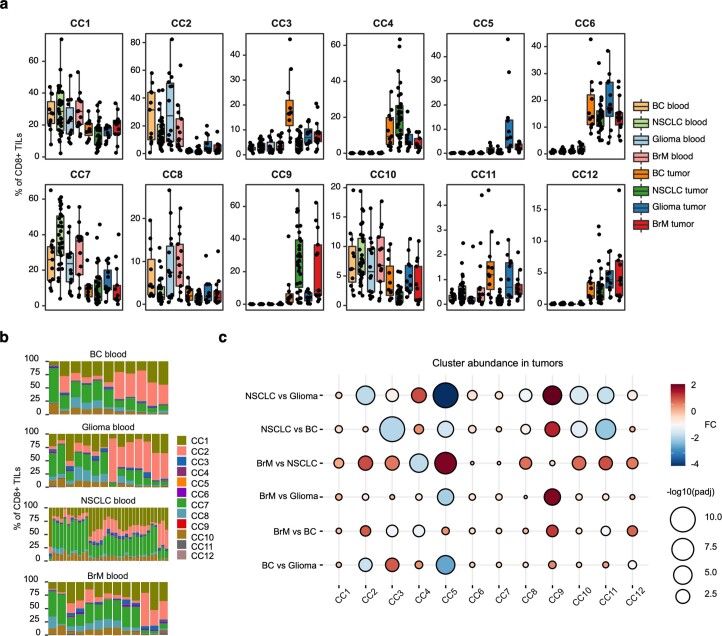
Abundance of pTRT in BrM and primary NSCLC is comparable, while the phenotypes are distinct. **a**, Abundance of each cytometry cluster (CC) in the blood and tumor of n_BC_ = 11, n_NSCLC_ = 30, n_glioma_ = 12, and n_BrM_ = 13 patients. **b**, Stacked bar plots showing the abundance of each CC in individual patient blood samples grouped by the disease. **c**, Balloon plot summarizing the differences in the abundance of each cluster in tumors of the four disease groups. Balloon size represents the -log10(adjusted p value), and the color indicates the fold change. For **a**, the box plots represent first and third quartiles with median as center; whiskers show 1.5 × interquartile range of the 25th and 75th percentiles. Source data

To further extend these findings, we performed high-dimensional FCM analyses on an additional cohort of TILs and blood T cells isolated from 12 individuals with glioma (8 *IDH* wild type and 4 *IDH* mutant), 13 individuals with BrM (10 NSCLC, 1 melanoma, 1 epidermoid cancer and 1 sarcoma), 11 individuals with primary BC and 33 individuals with NSCLC (Fig. 6b and Supplementary Tables 1a and 4). As above, we focused on CD8^+^ T cells. Following normalization of these data, we performed unsupervised clustering and defined 12 distinct cytometry clusters (CCs), several of which showed a clear tissue-specific prevalence (Fig. 6c and Extended Data Fig. 7a,b). Analysis of individual proteins allowed us to identify one pTRT cell cluster, CC9, expressing high levels of CD39, CXCL13, TIM3, PD1 and CD45RO (Fig. 6d). As expected, blood samples from all disease groups lacked cells of CC9, while in tumors, these cells were detected at varying frequencies (Fig. 6d,e and Extended Data Fig. 7a). In gliomas and primary BC tumors, CC9 cells comprised, on average, 5% of all CD8^+^ TILs, with only 1 BC tumor sample (1/11) displaying high CC9 abundance (Fig. 6e,f). By contrast, the vast majority of primary NSCLC tumors showed a high infiltration of CC9 cells, representing 25% of TILs on average. In BrM, we could again discriminate two groups of individuals, one with low CC9 (7/13) and one with high CC9 (6/13) abundance (Fig. 6f). The six BrM tumors with high CC9 abundance included one of one melanoma–BrM and five of ten NSCLC–BrM. Of note, in primary NSCLC and BrM tumors with a high proportion of CC9 (>30%), the abundance of these cells was similar (Fig. 6e,f). In general, CC9 was among the most variable across the disease groups (Extended Data Fig. 7c).

We independently confirmed these findings by manually gating for CD39^high^CCR7^low^CD8^+^ T cells and obtained very similar results (Fig. 6g,h). Finally, we compared the phenotype of CD39^high^CCR7^low^CD8^+^ TILs (pTRT cells) between the different tumor types. As only two of the glioma tumors had >100 pTRT cells, we excluded glioma samples from this specific analysis. We found several molecules enriched in primary NSCLC pTRT cells compared to BC and BrM pTRT cells, including ICOS, TIM3, CXCL13 and CD103 (Fig. 6i). By contrast, CD38, HLA-DR, GZMB and PD1 were highly expressed in BrM pTRT cells compared to pTRT cells in primary BC and NSCLC (Fig. 6i). Together, these analyses revealed that while the pTRT cell abundance was similar in primary NSCLC and BrM, particularly melanoma–BrM and NSCLC–BrM, the pTRT cell phenotype showed some underlying disease specificity.

## Discussion

In this study, we applied a diverse panel of orthogonal analyses to systematically and comprehensively profile T cells in primary and metastatic brain cancers. Our analysis revealed both phenotypic similarities and differences when interrogating TILs from BrM and glioma. The similarities were largely driven by T cell activation and differentiation, indicating the requirement of these processes for T cells to infiltrate brain tumor tissue. Similar observations have been made in several extracranial cancer types^26,39^. Importantly, our results indicate that in the majority of individuals with primary and metastatic brain cancer analyzed herein, activated TILs were likely not reactive against the tumor and were generally low in abundance. These pTRT cell-low tumors additionally showed signs of a non-inflamed ‘cold’ tumor microenvironment^40^ with relatively low levels of inflammatory and T cell-recruiting cytokines. Among potential mechanisms facilitating this environment are the physical exclusion of T cells from tumor nests through an aberrant vasculature, the engagement of different chemokine signaling pathways, including transforming growth factor-β (TGFβ) and CXCL12 (refs. ^41–44^), or an insufficient availability of tumor-specific/tumor-associated antigens^45^. Indeed, we found enrichment for TGFβ response genes in MDMs from pTRT cell-low BrM and glioma compared to in pTRT cell-high tumors (data not shown). Additionally, the majority of tumors classified as pTRT cell-low herein were either glioma or breast–BrM, and both are characterized by a relatively low somatic mutation prevalence^46^.

However, we identified a subset of individuals with BrM with a distinct CD8^+^ TIL phenotype, characterized by high *CXCL13* RNA expression, high CD39 protein levels and the enrichment of a dysfunctional gene signature. CD8^+^ TILs in these pTRT cell-high BrM were clonally expanded, showed little clonal overlap with matched blood CD8^+^ T cells and were located predominantly within tumor nests, suggesting specific tumor reactivity^16,35^. FCM and scRNA-seq analyses confirmed the presence of a CD8^+^ T cell population with a tumor reactivity phenotype in pTRT cell-high BrM, accounting for at least 30% of all CD8^+^ T cells. Approximately 50% of all analyzed BrM samples, and none of the gliomas, displayed such a pTRT cell-high tumor microenvironment. The infiltration of BrM tumors with pTRT cells has recently been reported^47^, however, without exploring the inter-individual heterogeneity. Here, we show that pTRT cell abundance differs substantially between primary and metastatic brain cancers and between individuals with BrM, even with the same disease subtype.

These findings naturally raise the question as to why the disease progresses in pTRT cell-high BrM. Several mechanisms that can suppress pTRT cell function have been described in other cancers^41^, including the accumulation of inhibitory cells in the tumor microenvironment, such as T_reg_ cells^48^ and suppressive myeloid cells^49^, as well as the release of soluble immunosuppressive molecules (for example, IDO1)^50^. These mechanisms could also play a role in pTRT cell-high BrM. Indeed, we found that T_reg_ cells were more prevalent in BrM than in gliomas, both in this study and in a recent analysis of the global brain immune cell landscape^5^, possibly resulting from increased tumor reactivity overall. Moreover, we determined that *IDO1*, which catabolizes tryptophan into the immunosuppressive metabolite kynurenine, is expressed at significantly higher levels in MDMs and MG isolated from pTRT cell-high BrM than those from pTRT cell-low BrM or glioma. However, it is important to note that different TAM subpopulations in pTRT cell-high BrM likely have distinct roles, immunosuppressive and inflammatory. In fact, we found higher expression of multiple T cell-recruiting molecules by MDMs and MG in pTRT cell-high BrM, along with genes involved in antigen presentation. Similar complex TAM phenotypes have been shown to support pTRT cells in ovarian, kidney, prostate and bladder cancer^37,38^. As such, several distinct subpopulations of TAMs may coexist in pTRT cell-high tumors, executing either immune suppression or inflammation, and likely depend on the spatial cues they are subject to within their local tumor microenvironment niche, as recent findings would suggest^51^.

In summary, this study has important implications for the clinical management of individuals with brain cancer. The abundance of *CXCL13*-expressing (CD39^+^) CD8^+^ T cells and the expression of *CXCL9* in the tumor microenvironment are robust predictors for ICB response in many different primary cancer types, including lung and triple-negative BC^22–24,52^. These factors may also indicate ICB susceptibility in the brain, and we found both to be significantly enriched in pTRT cell-high BrM. In addition, the proportion of CXCL13^+^CD39^+^CD8^+^ TILs in pTRT cell-high BrM was similar to that in primary NSCLC, one of the most responsive cancers to ICB^53^. This suggests that only individuals with pTRT cell-high, but not pTRT cell-low, tumors would potentially benefit from this treatment. While it remains an open question regarding how to evaluate the pTRT cell status of a brain tumor without surgical resection, our data provide the rationale to intensify research into minimal and non-invasive procedures to assess the quantity and quality of T cells within brain tumors.

## Methods

### Study approval

All experimental procedures performed on clinical tissue samples obtained from individuals were in accordance with the ethical standards of the institutional and national research committees and with the 1964 Helsinki Declaration and its later amendments or comparable ethical standards. Informed consent was obtained from all participants included in this study. The collection of tumor and non-tumor tissue and blood samples from individuals with brain disease at the Biobank of the Brain and Spine Tumor Center (BB_031_BBLBGT) of the Centre Hospitalier Universitaire Vaudois (Lausanne, Switzerland) was approved by the Commission Cantonale d’éthique de la Recherche sur l’être Humain (CER-VD, protocol PB 2017-00240, F25/99). The use of human samples from individuals with extracranial disease was approved by the Humanitas Clinical and Research Center Institutional Review Board (Milan, Italy) under the following protocols: lung cancer tissue and blood samples from individuals with NSCLC (1501) and BC tissue and blood from individuals with BC (ONC-OSS-02-2017). Tissue specimens were immediately collected from the operating room and processed as described below. All samples were fully anonymized. All available clinical information is included in Supplementary Table 1a.

### Clinical sample handling and processing

Clinical sample processing and preparation for conventional FCM, fluorescence-activated cell sorting and RNA-seq of sorted populations (bulk RNA-seq) were performed as described previously^27^, module 2. For cryopreservation, 0.5 × 10^6^–2 × 10^6^ cells were resuspended in ice-cold freezing medium containing 90% fetal bovine serum (Gibco) and 10% DMSO (Applichem), placed at −80 °C overnight in an alcohol-free freezing container (Corning, CoolCell) and transferred thereafter to liquid nitrogen for long-term storage. Cryopreserved samples were recovered by rapid thawing at 37 °C and were subsequently washed in complete medium comprised of DMEM/F12 (Thermo Fisher Scientific, 11320033), 10% fetal bovine serum and 1% penicillin–streptomycin (Thermo Fisher Scientific, 15070063).

### High-dimensional FCM

High-dimensional FCM was performed as described previously^54^. Dead cells were excluded from all analyses using Zombie Aqua (BioLegend). Fluorochrome-conjugated monoclonal antibodies were purchased from commercial vendors (Supplementary Table 5a). All reagents were titrated before use to determine optimal concentrations. Transcription factors and intranuclear molecules were measured in conjunction with a transcription factor buffer set (BD Biosciences). Samples were acquired using a FACSymphony A5 equipped with FACSDiva software version 8.0.1 (BD Biosciences). Electronic compensation was performed using single-stained controls prepared with antibody capture beads (BD Biosciences).

### Bioinformatic analysis environment

All bioinformatic analyses were performed within the R environment version 4.0.3 and Bioconductor version 3.12.

### Bulk RNA-seq, count matrix generation

Immune cell populations were sorted based on the markers and gating strategy described previously^5^. RNA extraction, library preparation and sequencing of sorted populations were performed at Genewiz Services (http://www.genewiz.com/) using the ultralow input RNA-seq service, which uses a poly(A) selection with enrichment for full-length transcripts. Paired-end sequencing of the libraries was performed using an Illumina HiSeq instrument with 2 × 150-base pair configuration. Raw sequencing reads were aligned to a reference human genome using STAR v2.7.7a^55^, and counts were quantified using RSEM v1.3.3 (ref. ^56^). Human genome version 38 was used with GENCODE v36 annotation. Raw counts of transcripts with the same gene symbol were pooled. Samples with <1,000,000 total counts were excluded.

RSEM counts of T cells were further processed to account for potential contamination with transcriptionally highly active tumor cells (sorted as CD45^–^ cells). A four-step process was applied for each disease group: (1) selecting 250 genes with highest absolute expression in CD45^–^ cells, (2) performing DEA between CD45^–^ cells and all T cells (blood and tumor) using the limma (RRID SCR_010943) package^57^, (3) identifying genes expressed at substantially higher levels in CD45^–^ cells than in T cells using very stringent cutoffs with a fold change of >3 and FDR of <0.001 and (4) generating overlap between DEGs from (3) and highly expressed tumor cell-specific genes from (1). The resulting genes (a total of 136) were removed from the count matrix (Supplementary Table 5b).

### Bulk RNA-seq, gene-focused analysis

All visualizations of counts are displayed as log_2_ (CPM) generated with the limma package. Annotated heat maps were generated with the pheatmap (RRID SCR_016418) package and show the expression of individual genes as *z* scores and are clustered using Euclidean distance. The 250 most variably expressed genes among all T cells were identified as those 250 genes with the largest variance in expression. For DEA, counts were first filtered using the filterByExpr function from the edgeR package (RRID SCR_012802)^58^ and normalized using the trimmed mean of the M values method (edgeR). The limma voom function was used to perform DEA. Significance cutoffs of a fold change of >2 or <−2 and an FDR of <0.05 were used.

### Bulk RNA-seq, pathway-centered analysis

Pathway enrichment in sample groups was analyzed by GSEA using the fgsea (RRID SCR_020938) package, considering pathways with a minimum size of 15 and maximum size of 500 genes. Gene ranks for fgsea were derived from the *t*-statistic of limma. Pathway enrichment in individual samples was analyzed by gene set variation analysis using the gsva method and log_2_ (CPM) as input. Hallmark, Gene Ontology biological process and C7 gene sets were obtained from MSigDB^59^ (version 7.2.1) using the msigdbr package. The C7 gene set collection was filtered to contain only pathways with ‘CD4’ or ‘CD8’ in their title.

### TCR analysis from bulk RNA-seq

To derive individual TCR β-chain sequences from our bulk RNA-seq data, the mixcr framework^60^ was used with default parameters. Briefly, the raw reads were cleaned from adaptor sequences using Trimmomatic^61^, and duplicated reads were removed with the clumpify function from bbmap (RRID SCR_016965). Processed reads were aligned against the reference V, D, J and C genes of the TCR (downloaded September 2019). Aligned reads were quantified, and identical reads were grouped into clonotypes. Here, only the TCR β-chain was used. The diversity of the TCR pools was evaluated with the vdj tools framework^62^, and the Chao estimate was used to determine the lower bound total diversity estimates (Chao 1 index).

### Sample and library preparation for single-cell sequencing

Tumor tissue and blood were prepared as for bulk RNA-seq analysis. Staining for T cell sorting was performed as for bulk RNA-seq. CD45^+^CD11B^–^CD3^+^CD4^+^ and CD45^+^CD11B^–^CD3^+^CD8^+^ cells were sorted into the same tube containing 8 µl of HBSS at 4 °C. Afterward, a maximum of 16,000 total T cells was loaded onto the 10x Chromium Controller following the manufacturer’s instructions. For the generation of gel beads in emulsion (GEM), a Chromium Next GEM single cell 5′ kit v2 (10x, 1000263) and the Chromium Next GEM Chip K single cell kit (10x, 1000287) were used. Gene expression (GEX) and TCR (VDJ) libraries were prepared using the library construction kit (10x, 1000190) and Chromium single cell human TCR amplification kit (10x, 1000252), respectively, following the manufacturer’s instructions.

Library quantity and quality were determined using Qubit fluorometric quantification (Thermo Fisher Scientific, Q32851) and high-sensitivity next-generation-sequencing fragment analysis (Agilent Technologies, DNF-474-0500). Sequencing was performed by Genewiz Services (http://www.genewiz.com/) on an Illumina NovaSeq6000 S4 flow cell using a 10x sequencing configuration. Targeted sequencing depth was >20,000 reads per cell for GEX and >5,000 reads per cell for VDJ libraries, respectively.

### Single-cell sequencing analysis

FastQ files of the GEX and VDJ libraries were aligned to the human reference genome GRCh38 2020-A (release July 7, 2020) and to the GRCh38 _alts_ensemble-5.0.0, respectively, using Cell Ranger software (version 6.0) from 10x Genomics. Downstream analysis was performed using Seurat (RRID SCR_016341) package version 4.0 (ref. ^63^) in R. For visualization, Seurat, dittoSeq^64^ and tidyverse (RRID SCR_019186) packages were used. For quality control, we retained only cells with <10% mitochondrial RNA and 250–3,000 total features. Samples were integrated with the SCTransform function^65^ using the day of sample sorting as the batch parameter. Clustering was performed with 31 dimensions in FindNeighbors and RunUMAP functions and a resolution of 0.18 in the FindClusters function. Cluster-specific genes were identified using the FindMarkers function with a fold change of >0.25 and at least 25% of cells in each cluster expressing the DEG using the Wilcoxon rank-sum test. Results were validated by receiver operating characteristic analysis in Seurat. Neoantigen-reactive CD8^+^ T cell gene signature scores were calculated using the AddModuleScore function. DEA between groups was performed using the FindMarkers function with the Poisson generalized linear model including batch as a covariant. VDJ libraries were processed with the scRepertoire package^66^. TCR similarity analysis was performed with the clonalOverlap function using the Morisita index.

### Prediction of TCR specificity against viral antigens

VDJmatch software version 1.3.1 was used to match TCR-β repertoires (generated by scRNA-seq) against the VDJdb (https://vdjdb.cdr3.net/) database compiling curated TCR sequences with known antigen specificity. The function match was used with default parameters to align TCR sequences for each CD8^+^ T cell cluster separately. Results were filtered using the vdjdb.score including matching sequences only with a vdjdb.score of ≥1. Unique matching viral antigens were counted and plotted.

### Computational analysis of high-dimensional FCM data

FCM standard 3.0 files were imported into FlowJo software version 9 (FlowJo). A conventional gating strategy was used to remove aggregates and dead cells. Viable CD3^+^CD8^+^ T cells were exported and used for downstream analysis. FlowJo software version 10.8.1 (FlowJo) was used for all analysis requiring manual gating. For unsupervised clustering analysis, samples with <100 CD8^+^ T cells were excluded. Remaining samples were downsampled to 1,500 cells with random sampling and imported into R using the flowCore and CATALYST packages (RRID SCR_002205). Data were transformed with arcsinh-transformation using a cofactor of 150. The flowSOM algorithm was used to cluster the cells^67^. For clustering, all markers were used with the exception of the live/dead dye, CD3, CD4, CD8 and FOXP3. Visual investigation of the different cluster numbers determined 12 as the most informative. UMAP projections were calculated with the runDR function from CATALYST.

### Sequential IF staining

The optimal cutting temperature-embedded tissues (Tissue-Tek, Sakura Finetek) were cut into 10-µm-thick slices, frozen and stored at −80 °C as outlined previously^27^. The integrity of the tissue was assessed using hematoxylin and eosin staining. Slides were air dried and fixed for 40 min at room temperature (RT) in 10% neutral buffered formalin (Fisher Scientific, Epredia 5701, 22-050-104). Afterward, the tissue was rehydrated in PBS (Gibco) by washing three times for 5 min each, followed by quenching in PBS and 10 mM glycine (Panreac Applichem ITW reagents, A1067) for 10 min at RT. The slides were then washed with PBS and 0.2% Tween (Applied Chemicals) two times for 5 min each, permeabilized with PBS and 0.2% Triton X-100 (Applied Chemicals) for 10 min and washed two times with PBS. Using an A-PAP pen, a hydrophobic circle was drawn in which the blocking solution was added for a 1-h incubation at RT inside a humidified chamber on a rocking platform. The blocking solution consisted of 1× PNB reagent (PerkinElmer, FP1012), 0.5% Tween, 10% donkey serum (Sigma-Aldrich, S30-M) and 2% bovine serum albumin (Jackson ImmunoResearch, 001-000-162) filtered through a 0.22-µm filter. At the end of this incubation period, the blocking buffer was replaced with the primary antibody solution (Supplementary Table 5a) in 0.22-µm-filtered antibody dilution buffer (1× PNB, 0.5% Tween and 10% normal donkey serum), and the tissue was incubated at 4 °C overnight in a humidified chamber. Alternatively, slides were labeled with the nuclear detection marker DAPI (Life Technologies, D1306) to record the autofluorescence of the tissue. The slides were then washed with PBS–Tween three times for 5 min each, and the fluorophore-conjugated secondary antibody solution containing DAPI in antibody dilution buffer was added, followed by an incubation period of 1 h in the dark at RT. Staining with secondary antibodies alone on the immediate next tissue section was used as a control. The slides were then washed six times for 10 min each in PBS–Tween and blocked for 1 h, followed by incubation with a directly conjugated antibody for 1 h at RT in a humidified chamber. The slides were then washed two times for 10 min each in PBS–Tween, followed by washing two times with PBS. SlowFade Diamond antifade mountant medium (Invitrogen, S36972) was added to each tissue, and a coverslip (Menzel-Gläser) was carefully mounted. The slides were scanned using the Axio scan.Z1. Following image acquisition, the coverslips were gently removed in PBS. The antibodies were eluted after a 5-min incubation with a freshly prepared tris(2-carboxyethyl)phosphine (TCEP)-based buffer (0.5 M glycine, 3 M guanidium chloride (Carl Roth, 0037.1), 2 M urea (Panreac Applichem ITW reagents, A1360) and 40 mM TCEP (Sigma, C4706) in double distilled water). Following this elution step, the slides were washed with PBS, and the sequential staining procedure was restarted by adding blocking buffer.

### Image processing and analysis

Before analysis was performed, the images were processed using ZEN Blue Software. The images were stitched, and the background was subtracted using the rolling ball method with a radius of 75. In addition, autofluorescence was removed by using the signal from the DAPI-only-stained tissue image. The images of multiple staining rounds were then aligned into one single image containing the information for all stained markers. Autofluorescence subtraction and alignment were performed using Python. Image quantification was performed using QuPath^68^. The aligned images were imported and divided into training (40%) and validation (60%) datasets. A 3 × 2 mm region of interest was randomly selected in each training image. Tumor tissue was detected using the pixel classifier RandomTrees trained by selecting the tumor versus non-tumor areas. Training was validated using the validation images and then applied to the complete dataset. A similar approach was used to identify the vessels. The PVN was created by expanding the vessels by 15 µm. Nuclear detection was performed with the StarDist protocol using a cell expansion of 3 (ref. ^69^). Finally, cell identification was performed using the object classifier RandomTrees for every marker separately. A composite classifier was generated from sequentially added single-marker classifiers to enable final cell identification and exported as a .csv file for downstream analysis with R.

The following were the cells of interest: (1) pTRT cells (CD45^+^CD3^+^CD8^+^ T cells double positive for CD103 and PD1), (2) non-pTRT cells (CD45^+^CD3^+^CD8^+^ T cells single positive or double negative for CD103 and PD1), (3) MG (CD45^+^CD68^+^ and/or P2RY12^+^CD49D^–^) and (4) MDMs (CD45^+^CD68^+^ and/or P2RY12^+^CD49D^+^).

Only cells with a diameter size of >4 µm and <12.5 µm and a detection probability of >0.65 were kept for the final analysis and visualization.

### Ex vivo T cell proliferation assay

Brain tumor tissue was prepared as for bulk RNA-seq. Fresh or thawed dissociated tumor cells (1 × 10^6^ cells per ml) were stained with 1 µl ml^–1^ CFSE (Thermo Fisher Scientific, C34554) in protein-free medium. The cells were stained for 20 min in the dark at RT. Following this incubation period, the remaining CFSE was quenched using complete medium. CFSE-labeled cells (1 × 10^5^) were plated on a flat-bottom, 96-well plate in 100 µl of complete medium. Where indicated, 40 µg ml^–1^ of the blocking anti-PD1 (clone EH12.2H7, BioLegend, 329926) or isotype control (BioLegend, 401401) was added. The cells were incubated at 37 °C and 5% CO_2_ for 96 h. Afterward, cells were stained with anti-CD45, anti-CD11B and anti-CD3 (ref. ^27^), and CFSE dilution in T cells was recorded. All antibodies used in this study are summarized in Supplementary Table 5a. Samples were acquired using a BD LSR Fortessa II, and data analysis was performed with FlowJo software version 10.8.1 (FlowJo).

### Statistics and reproducibility

No specific statistical method was used to predetermine sample size, but our cohort sizes are similar or larger than those reported in previous publications^5,7,47,51^. Normality of data distribution was not formally tested, and thus nonparametric statistical analyses were used. No randomization or blinding was performed due to the exploratory nature of the study. Only biological replicates were used for statistical analyses. Summary data are shown in the form of box plots, with the median as center, the 25th and 75th percentiles as the hinges and bounds of the box (first and third quartile) and the upper/lower whiskers extending from the hinge to the largest/smallest value but no further than 1.5× the interquartile range. For bulk RNA-seq, samples with <1,000,000 total counts were excluded. For TCR analysis from bulk RNA-seq, samples with <20 unique TCR sequences were excluded. For FCM analysis, samples with less than 100 cells in the analyzed population were excluded. For IF analysis, tissues with less than 30 total CD8^+^ T cells were excluded. Statistical analysis was performed with R version 4.0.3 or Prism version 9.3.1 using the test indicated within the corresponding figure caption or sections in the main text. Where relevant, samples were excluded before statistical analysis was performed. When more than two groups were compared, a correction for multiple testing was performed using the Benjamini–Hochberg method.

### Reporting summary

Further information on research design is available in the Nature Portfolio Reporting Summary linked to this article.

## Supplementary information

Reporting Summary

Supplementary Table 1 Supplementary Tables 1–5.

## Data Availability

RNA-seq count expression data and scRNA-seq data generated in this study can currently be visualized at https://joycelab.shinyapps.io/braintime/. Due to strict privacy protection, the raw RNA-seq data will be made available when possible. Future users can contact the corresponding author for access to the raw, unprocessed RNA-seq data, and those requests will then be individually reviewed by the relevant institutional committees. Gene signatures from the MSigDB can be found on the database website (http://www.gsea-msigdb.org/gsea/msigdb). Curated TCR sequences with known antigen specificity were obtained from the VDJdb database (https://vdjdb.cdr3.net/). Published gene signatures for neoantigen-reactive CD8^+^ T cells are provided in Supplementary Table 1c. Source data are provided with this paper. All other data supporting the findings of this study are available from the corresponding author upon reasonable request.

## Source data

Source Data Fig. 1 Statistical source data.
Source Data Fig. 2 Statistical source data.
Source Data Fig. 3 Statistical source data.
Source Data Fig. 4 Statistical source data.
Source Data Fig. 5 Statistical source data.
Source Data Fig. 6 Statistical source data.
Source Data Extended Data Fig. 1 Statistical source data.
Source Data Extended Data Fig. 2 Statistical source data.
Source Data Extended Data Fig. 3 Statistical source data.
Source Data Extended Data Fig. 4 Statistical source data.
Source Data Extended Data Fig. 6 Statistical source data.
Source Data Extended Data Fig. 7 Statistical source data.

## Code availability

No custom code was used or developed for the analyses presented in this study. Standard workflows and open-source R packages and software were used (Methods).
