# Supplementary material for: Phenotypic diversity of T cells in human primary and metastatic brain tumors revealed by multiomic interrogation
Source: Nat Cancer. 2023 May 22;4(6):908–24. doi: 10.1038/s43018-023-00566-3 (PMC10293012; doi:10.1038/s43018-023-00566-3)
Supplement: Supplementary file 1 — Reporting Summary [file 43018_2023_566_MOESM1_ESM.pdf]

Corresponding author(s): Johanna A. Joyce
Last updated by author(s): Apr 6, 2023

Reporting Summary

Nature Portfolio wishes to improve the reproducibility of the work that we publish. This form provides structure for consistency and transparency in reporting. For further information on Nature Portfolio policies, see our Editorial Policies and the Editorial Policy Checklist.

Statistics

For all statistical analyses, confirm that the following items are present in the figure legend, table legend, main text, or Methods section.

n/a	Confirmed	
☐	☒	The exact sample size (n) for each experimental group/condition, given as a discrete number and unit of measurement
☐	☒	A statement on whether measurements were taken from distinct samples or whether the same sample was measured repeatedly
☐	☒	The statistical test(s) used AND whether they are one- or two-sided<br>*Only common tests should be described solely by name; describe more complex techniques in the Methods section.*
☒	☐	A description of all covariates tested
☐	☒	A description of any assumptions or corrections, such as tests of normality and adjustment for multiple comparisons
☐	☒	A full description of the statistical parameters including central tendency (e.g. means) or other basic estimates (e.g. regression coefficient) AND variation (e.g. standard deviation) or associated estimates of uncertainty (e.g. confidence intervals)
☐	☒	For null hypothesis testing, the test statistic (e.g. F, t, r) with confidence intervals, effect sizes, degrees of freedom and P value noted<br>*Give P values as exact values whenever suitable.*
☒	☐	For Bayesian analysis, information on the choice of priors and Markov chain Monte Carlo settings
☒	☐	For hierarchical and complex designs, identification of the appropriate level for tests and full reporting of outcomes
☒	☐	Estimates of effect sizes (e.g. Cohen's d, Pearson's r), indicating how they were calculated

Our web collection on statistics for biologists contains articles on many of the points above.

Software and code

Policy information about availability of computer code

Data collection

Flow Cytometry data were collected on a BD Fortessa or BD FACSymphony A5 equipped with FACSDiva software version 8.0.1 (BD Biosciences). Cell sorting was performed on a BD AriaIII. Immune cell populations were sorted based on the markers and gating strategy described in (Maas, R. R. et al. Nature Protocols, 2021).
For bulk RNA sequencing, RNA extraction, library preparation and sequencing of sorted populations was performed at Genewiz Services (http://www.genewiz.com/) using the Ultra-Low Input RNA-Seq service which utilizes a Poly(A) selection with enrichment for full-length transcripts. Paired-end sequencing of the libraries was performed using the Illumina HiSeq instrument with 2x150 bp configuration. Raw sequencing reads were aligned to a reference human genome using STAR v2.7.7a and counts were quantified using RSEM v1.3.3. Human genome version 38 was used with GENCODE v36 annotation. Raw counts of transcripts with the same gene symbol were pooled. Samples with <1,000,000 total counts were excluded.
For single cell RNA sequencing, a maximum of 16,000 sorted T cells were loaded onto the 10x Chromium Controller following the manufacturer's instruction manual. For the generation of Gel Beads in Emulsion (GEM), Chromium Next GEM Single Cell 5' Kit v2 (10x, 1000263) and the Chromium Next GEM Chip K Single Cell Kit (10x, 1000287) were used. Gene expression (GEX) and T cell receptor (VDJ) libraries were prepared using the Library Construction Kit (10x, 1000190) and Chromium Single Cell Human TCR Amplification Kit (10x, 1000252), respectively, following the manufacturer's instruction manual. Library quantity and quality were determined using Qubit Fluorometric Quantification (Thermo Fisher Scientific, Q32851) and High Sensitivity Next-Generation-Sequencing Fragment Analysis (Agilent Technologies, DNF-474-0500). Sequencing was performed by Genewiz Services (http://www.genewiz.com/) on an Illumina NovaSeq6000 S4 Flow Cell using a 10x sequencing configuration. Targeted sequencing depth was >20'000 reads/cell for GEX and >5'000 reads/cell for VDJ libraries, respectively.
Immunofluorescence stainings of tissues were acquired with Zeiss Axio Scan.Z1 (Zeiss Germany) with ZEN software version 3.1 .

Data analysis

All bioinformatic analyses were performed within the R environment version 4.0.3 and Bioconductor version 3.12. Statistical analysis was performed in R (v. 4.0.3) or Prism version 9.3.1.

For bulk RNA Seq data, RSEM counts of T cells were processed to account for potential contamination with transcriptionally highly active tumor cells (sorted as CD45-negative cells). A four step process was applied for each disease group, respectively: a) Selecting 250 genes with highest absolute expression in CD45-negative cells; b) Performing differential expression analysis (DEA) between CD45-negative cells and all T cells (blood and tumor) using the limma (v. 3.46.0) (RRID:SCR_010943) package; c) Identifying genes expressed at substantially higher levels in CD45-negative cells compared to T cells using very stringent cutoffs with fold change > 3 and FDR < 0.001. d) Generating overlap between differentially expressed genes (DEG) from (c) and highly expressed tumor cell-specific genes from (a). The resulting genes (a total of 136) were removed from the count matrix (Supplementary Table 5b). Differential expression analysis of bulk RNA Seq data was performed with limma in R. Pathway enrichment in sample groups was analyzed by gene set enrichment analysis using the fgsea (v. 1.16.0) (RRID:SCR_020938) package, considering pathways with a minimum size of 15 and maximum size of 500 genes. Gene ranks for fgsea were derived from the t-statistic of limma. Pathway enrichment in individual samples was analyzed by gene set variation analysis (GSVA, v. 1.38.2) using the gsva method and variance stabilized counts as input.

To derive individual TCR beta chain sequences from our bulk RNAseq data, the mixcr (v. 3.0.12) framework (Bolotin, D. A. et al. 2015) was used with default parameters. Briefly, the raw reads were cleaned from adapter sequences using Trimmomatic (v. 0.39) (Bolger, A. M., Lohse, M. & Usadel, B. 2014) and duplicated reads were removed with the clumpify function from bbmap (v 39.01) (RRID:SCR_016965). Processed reads were aligned against the reference V, D, J and C genes of the T cell receptor (downloaded September 2019). Aligned reads were quantified and identical reads were summarized into clonotypes. Here, only the TCR beta chain was used and samples with less than 20 unique TCRs were excluded. The diversity of the TCR pools was evaluated with the vdj tools framework (v. 1.2.1) (Shugay, M. et al. 2015) and the Chao estimate was used to determine the lower bound total diversity estimates (Chao 1 index).

For the analysis of single cell RNAseq, FastQ files of the GEX and VDJ libraries were aligned to the human reference genome GRCh38 2020-A (release July 7, 2020) and to the GRCh38 _alts_ensemble-5.0.0, respectively, using cell ranger software (version 6.0) from 10x Genomics. Downstream analysis was performed using Seurat (RRID:SCR_016341) package version 4.0 in R. For visualization, Seurat, dittoSeq (v. 1.10.0) (Bunis, D. G. et al. 2020) and tidyverse (v. 1.3.0) (RRID:SCR_019186) packages were used. For quality control, we retained only cells with < 10% mitochondrial RNA and 250-3000 total features. Samples were integrated with the SCTransform function (Hafemeister, C. & Satija, R. 2019) using the day of sample sorting as the batch parameter. Clustering was performed with 31 dimensions in FindNeighbors and RunUMAP functions and a resolution of 0.18 in the FindClusters function. Cluster-specific genes were identified using the FindMarkers function with FC > 0.25 and at least 25% of cells in each cluster expressing the DEG utilizing the wilcox test. Results were validated by Receiver Operating Characteristic (ROC) analysis in Seurat. Signature scores were calculated using the AddModuleScore function. DEA between groups was performed using the FindMarkers function with the poisson generalized linear model including batch as a covariant. VDJ libraries were processed with the scRepertoire package (v. 1.8.0) (Borcherding, N. et al. 2020). TCR similarity analysis was performed with the clonalOverlap function using the Morisita index.The VDJmatch software version 1.3.1 was used to match TCR beta repertoires (generated by scRNA-seq) against TCR sequences with known antigen specificity. The function match was used with default parameters to align TCR sequences for each CD8+ T cell cluster separately. Results were filtered using the vdjdb.score including matching sequences only with vdjdb.score ≥ 1. Unique matching viral antigens were counted and plotted.

For the analysis of high-dimensional flow cytometry (FCM) data, Flow cytometry standard (FCS) 3.0 files were imported into FlowJo software version 9 (FlowJo LLC). A conventional gating strategy was used to remove aggregates and dead cells. Viable CD3+CD8+ T cells were exported and used for downstream analysis. FlowJo software version 10.8.1 (FlowJo LLC) was used for all analysis requiring manual gating. For unsupervised clustering analysis, samples with < 100 CD8+ T cells were excluded. Remaining samples were down-sampled to 1,500 cells with random sampling and imported into R using the flowCore (v. 2.2.0) and CATALYST (1.14.1) packages (RRID:SCR_002205). Data were transformed with arcsinh-transformation using a cofactor of 150. The flowSOM algorithm was used to cluster the cells (Van Gassen, S. et al. 2015). For clustering, all markers were used with the exception of the live/dead dye, CD3, CD4, CD8 and FOXP3. Visual investigation of the different cluster numbers determined 12 as the most informative parameter. UMAP projections were calculated with the runDR function from CATALYST.

For the analysis of sequential immunofluorescence (IF) staining, images were stitched and the background was subtracted using the rolling ball method with a radius of 75. In addition, autofluorescence was removed by using the signal from the DAPI-only stained tissue image. The images from multiple sequential staining rounds were then aligned into one single image containing the information for all stained markers. The autofluorescence subtraction and the alignment were performed using Python version 3.9.5. Image quantification was performed using QuPath version 0.3.2 (Bankhead, P. et al. 2017). The aligned images were imported and divided into training (40%) and validation (60%) datasets. Tumor tissue was detected using the pixel classifier trained by selecting the tumor vs non-tumor areas. The training was validated using the validation images and then applied to the complete dataset. Nuclear detection was performed with the StarDist protocol using a cell expansion of 3 (Schmidt, U. et al. 2018). A similar approach was used to identify the blood vessels. The perivascular niche (PVN) was created by expanding the vessels by 15µm. Finally, cell identification was performed using the object classifier for every marker separately. The training was validated before it was applied to the complete dataset. A composite classifier was generated from sequentially added single-marker-classifiers to enable final cell identification and exported as a .csv file for downstream analysis with R. Only cells with a diameter size of >4µm and <12.5µm, and a detection probability >0.65, were kept for the final analysis and visualization.

For manuscripts utilizing custom algorithms or software that are central to the research but not yet described in published literature, software must be made available to editors and reviewers. We strongly encourage code deposition in a community repository (e.g. GitHub). See the Nature Portfolio guidelines for submitting code & software for further information.

Data

Policy information about availability of data

All manuscripts must include a data availability statement. This statement should provide the following information, where applicable:

- Accession codes, unique identifiers, or web links for publicly available datasets
- A description of any restrictions on data availability
- For clinical datasets or third party data, please ensure that the statement adheres to our policy

RNA-seq count expression data and single-cell RNAseq data generated in this study can currently be visualized at:
http://sib-pc17.unil.ch:3880/BrainTIME_v2/
Username:JoyceLab
Password: braintimev2_joycelab_2022
Due to patient privacy protection, the raw RNA-seq data will be made available upon request. Gene signatures from the MSigDB can be found on the database website (http://www.gsea-msigdb.org/gsea/msigdb). Curated TCR sequences with known antigen specificity were obtained from the VDJdb database (https://vdjdb.cdr3.net/). Published gene signatures for neoantigen-reactive CD8+ T cells are provided in Supplementary Table 1c. Source data have been provided as Source Data files. All other data supporting the findings of this study are available from the corresponding author upon reasonable request.

Human research participants

Policy information about studies involving human research participants and Sex and Gender in Research.

Reporting on sex and gender

The sex information from all participants has been collected and consent has been obtained for reporting. The sex information was obtained based on self-report.

Population characteristics

Details on human samples can be found in Supplementary Table 1a and 4.
The complete brain cancer patient cohort is comprised of n=84 patient specimens as follows:
Glioma n=36 (53% male and 47% female, median age: 56 years)
Brain metastasis: n=48 (42% male and 58% female, median age: 62 years)

The complete cohort of patient with extra-cranial tumors is comprised of n=45 patient specimens as follows:
Non-small cell lung cancer: n=34 (53% male and 47% female, mean age: 69.5 years)
Breast cancer: n=11 (100% female, median age: 66.18 years)

Recruitment

Samples from brain cancer patients were collected at the Centre Hospitalier Universitaire Vaudois (CHUV, Lausanne, Switzerland).
Samples from patients with extra-cranial cancers were collected at Humanitas Hospital (Milan, Italy).

Ethics oversight

The collection of tumor and non-tumor tissue and blood samples from patients with brain disease at the Biobank of the Brain and Spine Tumor Center (BB_031_BBLBGT) of the Centre Hospitalier Universitaire Vaudois (CHUV, Lausanne, Switzerland) was approved by the Commission Cantonale d'éthique de la recherche sur l'être humain (CER-VD, protocol PB 2017-00240, F25 / 99). The use of human samples from patients with extracranial disease was approved by the Humanitas Clinical and Research Center Institutional Review Board (Milan, Italy) under the following protocols: lung cancer tissue and blood samples from patients with non-small cell lung cancer (1501) and breast cancer tissue and blood from patients with breast cancer (ONC-OSS-02-2017).

Note that full information on the approval of the study protocol must also be provided in the manuscript.

Field-specific reporting

Please select the one below that is the best fit for your research. If you are not sure, read the appropriate sections before making your selection.

☒ Life sciences ☐ Behavioural & social sciences ☐ Ecological, evolutionary & environmental sciences

For a reference copy of the document with all sections, see nature.com/documents/nr-reporting-summary-flat.pdf

Life sciences study design

All studies must disclose on these points even when the disclosure is negative.

Sample size

No specific statistical method was used to predetermine sample size, but our cohort sizes are similar or larger than those reported in previous publications studying primary and metastatic human brain cancer which were able to show statistically significant results with their cohorts.

Data exclusions

Bulk RNAseq: Samples with <1,000,000 total counts were excluded. For TCR analysis, samples with <20 unique TCR sequences were excluded.
Flow cytometry: Samples with less than 100 cells in the analyzed population were excluded.

	Immunofluorescence: Tissues with less than 30 CD8+ T cells were excluded.<br>Samples were excluded before statistical analysis was performed.
Replication	Each condition/group in the RNAseq, flow cytometry, immunofluorescence staining, and functional analysis contained > 3 independent patient samples. The exact sample sizes are indicated in the figure legends.<br>The in vitro assay was repeated multiple times with independent tissue donors.<br>Immunofluorescence staining images are representatives of multiple replicates from the respective experimental group.<br>While individual bulk RNAseq, single cell RNAseq, flow cytometry, immunofluorescence, and ex vivo functional assays could not all be repeated due to limited size of available material, the core results from each method are completely in line with each other. Thus, each method represents an independent validation of the reproducibility of the main results in this study.
Randomization	Does not apply to this study because it is an exploratory and retrospective analysis of surgically resected brain tumor samples. Tissue was obtained and analyzed as it became available and was sufficient in size.
Blinding	Since this study is an exploratory and retrospective analysis of surgically resected tumor samples, blinding was not required. Moreover, bio-informatics analysis was performed using automated analysis tools described here and in the Methods section.

Reporting for specific materials, systems and methods

We require information from authors about some types of materials, experimental systems and methods used in many studies. Here, indicate whether each material, system or method listed is relevant to your study. If you are not sure if a list item applies to your research, read the appropriate section before selecting a response.

Materials & experimental systems

n/a	Involved in the study
☐	☒ Antibodies
☒	☐ Eukaryotic cell lines
☒	☐ Palaeontology and archaeology
☒	☐ Animals and other organisms
☒	☐ Clinical data
☒	☐ Dual use research of concern

Methods

n/a	Involved in the study
☒	☐ ChIP-seq
☐	☒ Flow cytometry
☒	☐ MRI-based neuroimaging

Antibodies

Antibodies used

All antibodies used in this study are summarized in Supplementary Table 5a.
Information below includes: ANTIBODY; FLUOROCHROME; DILUTION; CLONE; SOURCE; IDENTIFIER; RRID(where available).

Flow cytometry:
Anti-human CD8 BUV805 1:166 SK1 BD Biosciences Cat # 564912 RRID:AB_2744465
Anti-human CD45RO BV570 1:40 UCHL1 BioLegend Cat # 304226 RRID:AB_2563818
Anti-human CD4 BUV615 1:3,333 SK3 BD Biosciences Cat # 624297 N/A
Anti-human CD3 BUV496 1:40 UCHT1 BD Biosciences Cat # 564809 RRID:AB_2744388
Anti-human CCR7 PE-CF594 1:40 150503 BD Biosciences Cat # 562381 RRID:AB_11153301
Anti-human CD28 BV785 1:166 CD28.2 BioLegend Cat # 302950 RRID:AB_2632607
Anti-human CD25 BUV563 1:160 2A3 BD Biosciences Cat # 612918 RRID:AB_2870203
Anti-human CD69 BUV737 1:322 FN50 BD Biosciences Cat # 564439 RRID:AB_2722502
Anti-human CD127 PE-Cy5 1:40 eBioRDR5 eBioscience Cat # 15-1278-42 RRID:AB_2043801
Anti-human PD-1 BV480 1:27 EH12.1 BD Biosciences Cat # 566112 RRID:AB_2739514
Anti-human HLA-DR BUV661 1:166 G46-6 BD Biosciences Cat # 565073 RRID:AB_2722500
Anti-human CD38 BV711 1:166 HIT2 BioLegend Cat # 303528 RRID:AB_2563811
Anti-human CD103 BV421 1:166 Ber-ACT8 BioLegend Cat # 350213 RRID:AB_2563513
Anti-human CD161 BV605 1:20 HP-3G10 BioLegend Cat # 339916 RRID:AB_2563607
Anti-human Granzyme B APC-R700 1:80 GB11 BD Biosciences Cat # 561016 RRID:AB_2033973
Anti-human Granzyme K PE 1:166 GM6C3 Santa Cruz Cat # sc-56125 PE RRID:AB_2263772
Anti-human T-bet PE-Cy7 1:666 4-B10 eBioscience Cat # 25-5825-82 RRID:AB_11042699
Anti-human ICOS BUV395 1:80 DX29 BD Biosciences Cat # 564777 RRID:AB_2738946
Anti-human TIM3 BV650 1:160 7D3 BD Biosciences Cat # 565564 RRID:AB_2722547
Anti-human CD39 APC-Cy7 1:80 A1 BioLegend Cat # 328226 RRID:AB_2571981
Anti-human CXCL13 APC 1:80 53610 Thermo Fisher Cat # MA5-23629 RRID:AB_2610225
Anti-human Ki67 FITC 1:20 B56 BD Biosciences Cat # 556026 RRID:AB_396302
Anti-human FoxP3 PE-Cy5.5 1:40 PCH101 eBioscience Cat # 35-4776-42 RRID:AB_11218682
anti-human-CD45 AF700 1:640 HI30 BioLegend Cat#304024 RRID:AB_493761
anti-mouse/human-CD11B BV421 1:1280 M1/70 BioLegend Cat#101251 RRID:AB_2562904
anti-human-CD66B PE 1:200 G10F5 BioLegend Cat#305106 RRID:AB_2077857
anti-human-CD14 AF488 1:640 HCD14 BioLegend Cat#325610 RRID:AB_830683
anti-human-CD16 BUV737 1:640 3G8 BD Biosciences Cat#612786 RRID:AB_2833077
anti-human-CD49D APC 1:320 9F10 BioLegend Cat#304308 RRID:AB_2130041
anti-human-CD11C BV605 1:320 3.9 BioLegend Cat#301636 RRID:AB_2563796
anti-human-HLA-DR BV711 1:320 L243 BioLegend Cat#307644 RRID:AB_2562913

anti-human-CD3 PerCP/Cy5.5 1:80 HIT3a BioLegend Cat#300328 RRID:AB_1575008
anti-human-CD4 BV650 1:200 OKT4 BioLegend Cat#317436 RRID:AB_2563050
anti-human-CD25 PE 1:80 BC96 BioLegend Cat#302606 RRID:AB_314276
anti-human-CD127 BV510 1:160 A019D5 BioLegend Cat#351332 RRID:AB_2562304
anti-human-CD8A PE/Cy7 1:320 HIT8a BioLegend Cat#300914 RRID:AB_314118
anti-human-CD19 BUV563 1:320 SJ25C1 BD Biosciences Cat#612916 N/A
anti-human-CD56 PE/Dazzle 1:640 HDC56 BioLegend Cat#318348 RRID:AB_2563564
Anti-human CD45RO BV650 1:200 UCHL1 BioLegend Cat#304231 RRID:AB_2561359
Anti-human CD45RA BV605 1:400 HI100 BioLegend Cat#304133 RRID:AB_11126164

Immunofluorescence staining
Anti-human CD45 not-conjugated 1:100 polyclonal LSBio Cat# LS-B14248-300 RRID:AB_2889893
Anti-human CD103 not-conjugated 1:100 EPR22590-27 Abcam Cat# ab224202 RRID:AB_2891141
Anti-human CD8 not-conjugated 1:100 4B11 Bio-rad Cat# MCA1817T RRID:AB_323534
Anti-human CD3 AF647 1:50 UCHT1 BioLegend Cat# 300416 RRID:AB_389332
Anti-human CD31 not-conjugated 1:100 polyclonal R&D Systems Cat# PA5-96055 RRID: AB_2549792
Anti-human PD1 AF647 1:50 NAT105 BioLegend Cat# 367419 RRID:AB_2721353
Anti-human P2RY12 not-conjugated 1:600 polyclonal Sigma-Aldrich Cat# HPA014518 RRID:AB_2669027
Anti-human CD68 not-conjugated 1:100 KP1 Abcam Cat# ab955 RRID:AB_307338
Anti-human CD49D not-conjugated 1:100 PS/2 BioXCell Cat# BE0071 RRID:AB_1107657
Anti-human Pan-Cadherin not-conjugated 1:100 polyclonal Abcam Cat# ab16505 RRID:AB_443397
Donkey anti-goat AF488 AF488 1:500 polyclonal Invitrogen Cat# A32814 RRID:AB_2762838
Donkey anti-rabbit AF555 AF555 1:500 polyclonal Invitrogen Cat# A-32794 RRID:AB_2762834
Donkey anti-rat IgG H&L AF647 AF647 1:500 polyclonal Abcam Cat# ab150155 RRID:AB_2813835
Donkey anti-mouse IgG (H+L) AF647 AF647 1:500 polyclonal Invitrogen Cat# A-31571 RRID:AB_162542
Donkey anti-rabbit IgG (H+L) AF755 AF755 1:500 polyclonal Invitrogen Cat# SA5-10043 RRID:AB_2556623

Ex vivo treatment
Ultra-LEAF™ Purified anti-human PD1 (Mouse IgG1) not-conjugated 40ug/ml EH12.2H7 BioLegend Cat# 329926 RRID:AB_11147365
Purified Mouse IgG1, κ Isotype Ctrl not-conjugated 40ug/ml "
MG1-45" BioLegend Cat# 401401 RRID:AB_2801452

Validation

All antibodies were validated by the respective vendors. Relevant information on antibody validation can be found on the manufacturer's websites using the catalog number which is provided for each antibody used and listed in the section above.

Antibodies purchased from BioLegend for flow cytometry, immunofluorescence staining, and the functional assays were validated as followed: “Specificity testing of 1-3 target cell types with either single- or multi-color analysis (including positive and negative cell types). Once specificity is confirmed, each new lot must perform with similar intensity to the in-date reference lot. Brightness (MFI) is evaluated from both positive and negative populations. Each lot product is validated by QC testing with a series of titration dilutions.” (https://www.biolegend.com/en-us/quality/quality-control).
Antibodies purchased from BD Bioscience for flow cytometry were validated as followed: “The specificity is confirmed by using multiple applications that may include a combination of flow cytometry, immunofluorescence , immunohistochemistry or western blot to test a combination of primary cells, cell lines or transfectant models. All flow cytometry reagents are titrated on the relevant positive or negative cells.” (https://www.bdbiosciences.com/en-ch/products/reagents/flow-cytometry-reagents/research-reagents/quality-and-reproducibility).
Antibodies purchased from eBioscience/ Thermo Fisher were validated as followed: “[...] antibody has been pre-titrated and tested by flow cytometric analysis of normal human peripheral blood cells.” (https://www.thermofisher.com/ch/en/home/life-science/antibodies/invitrogen-antibody-validation.html).
Antibodies purchased from Abcam for immunofluorescence staining were validated as followed: “Antibody specificity is confirmed by looking at cells that either do or do not express the target protein within the same tissue. [...] We then check the protein expression by IHC/ICC to see if it has the expected cellular localization. [...] We use a variety of methods, including staining multi-normal human tissue microarrays (TMAs), multi-tumor human TMAs [...]” (https://www.abcam.com/primary-antibodies/how-we-validate-our-antibodies#IHC%20and%20ICC).
Antibodies purchased from R&D Systems for immunofluorescence staining were validated as followed: “ [...] We are continuously testing our products with knockout cell lines to ensure our antibodies are detecting the correct target. [...]” (https://www.rndsystems.com/quality/antibodies-built-for-reproducibility).
The specificity of the anti-human P2RY12 antibody from Sigma-Aldrich was validated with the companies’ standard method and additionally with orthogonal RNAseq: “We [...] test in as many additional immunodetection applications as practical in samples chosen to be relevant to the intended use of the product. These include immunohistochemistry, immunocytochemistry (ICC), Western blot, ELISA, immunoprecipitation, and more. [...] What is demonstrated is a direct comparison of mRNA expression level and sample staining.” (https://www.sigmaaldrich.com/CH/en/technical-documents/technical-article/protein-biology/immunohistochemistry/antibody-enhanced-validation).

In addition to the validation by the distributors, antibodies were titrated in our lab for the use with brain tumor samples. Full-minus-one (FMO) controls were used in flow cytometry and secondary-only controls were used in immunofluorescence staining analysis. An Mouse IgG1, κ Isotype control antibody was used in the ex-vivo functional assay.

Flow Cytometry

Plots

Confirm that:

☒ The axis labels state the marker and fluorochrome used (e.g. CD4-FITC).

☒ The axis scales are clearly visible. Include numbers along axes only for bottom left plot of group (a 'group' is an analysis of identical markers).

☒ All plots are contour plots with outliers or pseudocolor plots.

☒ A numerical value for number of cells or percentage (with statistics) is provided.

Methodology

Sample preparation	Sample preparation was as described previously in (Klemm et al., 2020; Maas et al., 2021). Tissue was macrodissected and enzymatically digested with the Brain Tumor Dissociation Kit (P) for glioma samples and with the Tumor Dissociation Kit for BrM samples, both purchased from Miltenyi, and using an OctoMacs dissociator according to manufacturers instructions. Cell suspensions were filtered through a 70 µm mesh filter and, for glioma samples only, incubated with Myelin Removal Beads for 15 mins at 4 °C prior to magnetic separation by filtering through LS Columns (Miltenyi). Red blood cell lysis was performed using the RBC buffer from Biolegend according to the manufacturer's instructions. In between individual steps, samples were washed with FACS buffer (PBS + 0.5 % BSA + 2 mM EDTA) and centrifuged for 10 mins at 300 g. Dead cells were labeled by incubation with the Zombie NIR solution (BioLegend) for 10 mins at RT. Samples were subsequently incubated with Human TruStain FcX Fc receptor blocking solution (BioLegend) for 10 mins at RT, followed by antibody incubation at 4 °C, 15 mins. Samples were washed and resuspended in FACS Buffer + 1:100 EDTA (0.25 M) and kept on ice until flow cytometric analysis or sorting.
Instrument	Flow Cytometry: BD Fortessa and FACSymphony A5<br>FACS Sort: Aria III
Software	BD Facs Diva software. Downstream analysis with R.
Cell population abundance	Post-sort reanalyses were performed with initial sorts which revealed >90% purity of the cell populations and is illustrated in Extended Data Fig. 1b and 2a. Median fluorescence intensity (MFI) analysis was performed on samples with at least 100 cells in the analyzed population.
Gating strategy	The gating strategy for flow cytometry and cell sorting was as previously described (Klemm et al., 2020) and can additionally be found in detail in this study in Extended Data Fig. 1b, 2a, 2c, 5d and Figure 3a, 3i, 6g.<br>FSC and SSC were used for identification of cells of interest. FSC-A, FSC-W and FSC-H were used to exclude doublets. Live/dead cell staining was performed with Zombie NIR Fixable Viability Kit (BioLegend).

☒ Tick this box to confirm that a figure exemplifying the gating strategy is provided in the Supplementary Information.
